# Multiple Mycotoxins Determination in Food by LC-MS/MS: An International Collaborative Study

**DOI:** 10.3390/toxins11110658

**Published:** 2019-11-12

**Authors:** Thomas Bessaire, Claudia Mujahid, Pascal Mottier, Aurélien Desmarchelier

**Affiliations:** Nestlé Research, Route du Jorat 57, Vers-chez-les-Blanc, 1000 Lausanne 26, Switzerland; claudia.mujahid@rdls.nestle.com (C.M.); pascal.mottier@rdls.nestle.com (P.M.); aurelien.desmarchelier@rdls.nestle.com (A.D.)

**Keywords:** LC-MS/MS, mycotoxins, collaborative study, isotopic dilution, compliance, infant food

## Abstract

An intercollaborative study was organized to evaluate the performance characteristics of a liquid chromatography tandem mass spectrometry procedure for the simultaneous determination of 12 mycotoxins in food, which were ochratoxin A, aflatoxins B1, B2, G1, G2, and M1, deoxynivalenol, zearalenone, fumonisins B1 and B2, and T-2 and HT-2 toxins. The method combined the simplicity of the QuEChERS (Quick, Easy, Cheap, Efficient, Rugged and Safe) approach with the efficiency of immunoaffinity column cleanup (the step used to enhance sensitivity and sample cleanup for some matrices only). Twenty-three entities were enrolled and were European reference laboratories for mycotoxin analysis, U.S. and European service laboratories, and Nestlé laboratories. Each participant analyzed 28 incurred and/or spiked blind samples composed of spices, nuts, milk powder, dried fruits, cereals, and baby food using the protocol given. Method performances were assessed according to ISO 5725-2. Relative standard deviations of repeatability and reproducibility and trueness values for each of the 115 mycotoxin/sample combinations ranged from 5% to 23%, 7% to 26%, and 85% to 129%, respectively, in line with requirements defined in EC 401/2006. The overall set of data gathered demonstrated that the method offered a unique platform to ensure compliance with EC 1881/2006 and EC 165/2013 regulations setting maximum limits for mycotoxins in food samples, even at low regulated levels for foods intended for infants and young children. The method was applicable regardless of the food, the regulated mycotoxin, and the concentration level, and thus is an excellent candidate for future standardization.

## 1. Introduction

Mycotoxins are a group of toxic chemical substances produced by filamentous fungi (molds) that commonly grow on a number of food commodities such as cereals, nuts, spices, fruits, oil seeds, or coffee. These toxins can be produced before harvest in the crop and even after harvest if climate conditions are favorable for further fungal growth. Mycotoxins are undoubtedly one of the most severe food safety hazards amplified by global climate change. Indeed, the ability of fungi to produce mycotoxins is largely influenced by temperature, precipitation, relative humidity, and stress conditions in the plants. For instance, aflatoxin-producing fungi (mainly *Aspergillus flavus* and *Aspergillus parasiticus*), usually more prevalent in tropical and sub-tropical regions, are expected to be found in areas such as southern and eastern Europe or the USA, where temperatures >30 °C (close to the optimum for aflatoxin production) may become usual [1,2].

Among several hundreds of mycotoxins identified so far, a few are of concern from a food safety perspective. To protect consumer health, maximum levels (MLs) for mycotoxins in foodstuffs have been established worldwide. In particular, the European Union legislation (often considered as the most stringent one) has established MLs for aflatoxins (AFLAs), ochratoxin A (OTA), zearalenone (ZEN), fumonisins (FBs), and deoxynivalenol (DON) (EC 1881:2006 [3]) and recently indicative levels for T-2 and HT-2 toxins (EC 165/2013 [4]) in a broad range of food commodities. Lower MLs have been also established for food intended for infants and young children.

International organizations such as ISO (International Organization for Standardization), CEN (European Committee for Standardization), or AOAC International have continued gathering experts over the years to develop internationally recognized analytical standards. The goal is to avoid discrepancies in results that may arise from the use of different analytical approaches, with the risk to distorted global food trade. Today, there are 72 official methods available from these organizations for the monitoring of mycotoxins in food (Table 1). These methodologies are often limited to a single compound or to certain family of mycotoxins only and generally validated for a single food category. Indeed, current regulations were established on toxicological data from studies taking into account only one mycotoxin exposure at a time and did not consider the combined effects of mycotoxins [5]. Noteworthy, the co-occurrence of mycotoxins has been extensively described over the last decade. In 2016, data from 107 publications were compiled to summarize the findings on mycotoxins and their co-occurrence in various foods and feeds from all over the world [5]. AFLAs + FBs, DON + ZEN, AFLAs + OTA, and FBs + ZEN were the most observed combinations. Another study on barley and wheat (*n* = 72 and *n* = 83, respectively) evidenced that among the mycotoxin-positive samples, 70% of barley samples and 54% of wheat samples were co-contaminated with at least two mycotoxins [6]. The need to develop methods able to screen several mycotoxins at once was justified in a large-scale global survey in feed where mycotoxin co-contamination was common [7]. Concentrations of aflatoxin B1 (AFB1), ZEN, FBs, OTA, DON, and T-2 toxin were analyzed in 74,821 samples of feed and feed raw materials (e.g., maize, wheat, soybean) collected from 100 countries from 2008 to 2017. In total, a large fraction of samples (64%) was co-contaminated with more than two mycotoxins, whilst 88% of the samples were contaminated with at least one mycotoxin. In that regard, moving from single residue analysis toward “multi-analyte and multi-matrix” ones is of interest to speed up efficiently and rationalize mycotoxin analysis in high-throughput routine environments.

Most of the current reference methods still make use of non-confirmatory approaches for quantitative analysis of mycotoxins, e.g., TLC, HPLC-UV, and HPLC-FLD. Use of IAC cleanup often compensates for the lack of specificity of these detection techniques, as authorized in EC 2002/657 [8]. However, LC-MS/MS is currently considered as the state-of-the-art technique to analyze hundreds of contaminants (pesticides, veterinary drugs, etc.) in various food commodities, including mycotoxins, as emphasized in a recent review [9]. The first official LC-MS based methodologies for the determination of mycotoxins in foods were published in 2017 by the CEN and are limited to ZEN [10], and T-2/HT-2 toxins’ [11] determination. Indeed, the need for standardized LC-MS methods for mycotoxins’ determination was highlighted only recently by regulatory authorities and scientific advisory bodies, triggering the CEN organization to establish European standards within the M/520 standardization mandate [12]. Among the several LC-MS methods still under development or at the final approval stage at CEN, only two are multi-mycotoxin methods for food analysis [13]. The first one is devoted to the screening of OTA, AFB1, DON, ZEN, FB1, FB2, T-2, and HT-2 toxins and excludes foods for infants and young children from the scope. The second one does not include regulated aflatoxins, zearalenone, and fumonisins and is limited to cereals and cereal based products. Such limited analyte and/or matrix scopes are constraints for food business operators willing to use efficient standard protocols to ensure the safety and compliance of a broad range of food commodities.

To fill this gap on our side, an analytical method was developed and internally validated in 2013 [14] before its deployment in 10 Nestlé Quality Assurance Centers (NQACs, control laboratories) in Brazil, Chile, the USA, France, Italy, Poland, Russia, China, Singapore, and India. The procedure enables the quantitative LC-MS/MS determination of regulated mycotoxins (OTA, AFB1, AFB2, AFG1, AFG2, AFM1, DON, ZEN, FB1, FB2, and T-2 and HT-2 toxins) in a broad range of food items including cereals and cereal based baby foods (infant cereals, biscuits), spices, nuts, coffee, tea, cocoa, vegetable oils, dried fruits, infant formula, dairy products, feed/pet food, etc. This routine method has the advantage of offering a unique platform to ensure full compliance with the EC 1881/2006 regulation [3], even at low regulated levels for foods intended for infants and young children. Its sample preparation combines the simplicity of the QuEChERS approach (widely used for pesticide residues analysis; European Norm EN 15662:2018 [15]) with the efficiency of IAC cleanup (this step being used for some matrices only). Quantification is performed by the isotopic dilution approach using ^13^C-labeled mycotoxins as internal standards (ISTD). The collection of thousands of validation data over time [16], the high number of analyses conducted each year (15,000 samples analyzed in 2017 [17]), and the regular enrollment of NQACs to internal (under ISO 17043:2010 accreditation [18]) and external proficiency tests have demonstrated the high robustness of this method.

In this context, we set up an international collaborative study to support its standardization. Twenty-three (23) laboratories from authorities and private sectors were involved in this study, representing entities from 14 countries. This collaborative study was organized according to the AOAC International guideline [19], and statistical evaluation was performed following ISO 5725-2 document [20]. The present paper summarizes the overall set of data gathered and demonstrates the method’s applicability over a broad range of concentrations across different food categories, including foods for infants and young children.

## 2. Results and Discussion

### 2.1. Samples and Homogeneity Testing

Providing blank matrices to be further spiked by each participant as done in previous studies [21] was not considered to avoid potential analytical bias introduced by operators. Alternatively, the preference was to use blind duplicates of former proficiency test samples still available as quality control (QC) materials (paprika, black pepper, almonds, hazelnuts, dried raisins, dried figs, wheat, maize (2), milk powder (2), maize (2), rice, and wheat based infant cereals samples). The homogeneity of such materials was extensively tested by their providers. However, since the study samples were repacked from 200-g aluminum bags to 25-g units before shipping, a small-scale testing was performed (six replicates from six different units for each sample) to ensure sample homogeneity. The resulting coefficients of relative standard deviation of repeatability RSD_r_ ranged from 0.5% to 12%, in line with internal validation data, evidencing the satisfactory homogeneity of the set of samples. The exception was a maize sample with unacceptable RSD_r_ for ZEN and HT-2, at 40% and 21%, respectively. The particle size distribution of this material was heterogeneous (evidence by visual inspection), which led us to re-open and merge all individual units. The resulting bulk material (ca. 2 kg) was ground in our laboratory by means of a cryogenic grinder (SPEX 6875D Freezer/Mill, Stanmore, U.K.) and further dispatched again into 25-g units. Subsequent homogeneity testing (duplicate analysis of six different units) successfully validated this new preparation with a global decrease of RSD_r_ for the twelve mycotoxins (ZEN and HT-2 at 6.7 and 10.6%, respectively).

### 2.2. Participants Instrumental Method Setup

**Figure 1:**
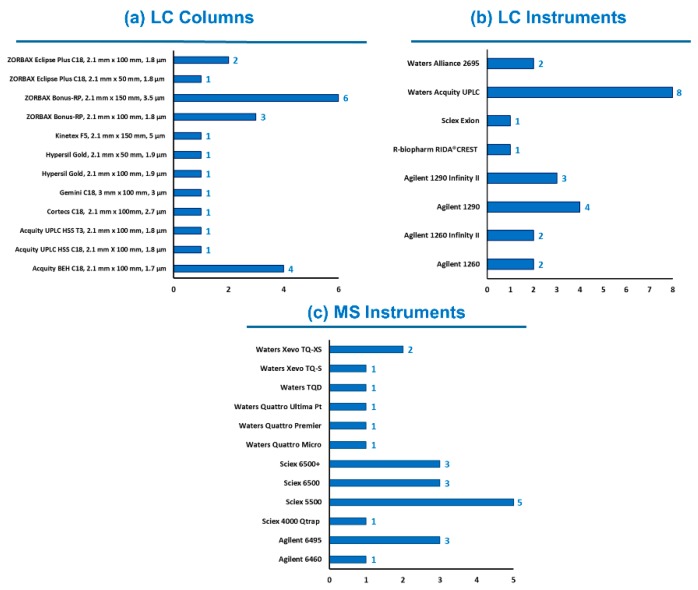
Instrumental setup used by the participating laboratories (*n* = 23).

Knowing the diversity of laboratory equipment available worldwide, participants were free to select their instrumental setup, meaning choice of LC columns, mobile phases, MS parameters, etc. Typical LC-MS/MS conditions were provided by the study director for information purposes only. The use of HR-MS instruments was accepted, but none of the twenty-three participants reported its usage. As shown in Figure 1, 13 different LC columns and 11 models of MS instruments were engaged. Mobile phases and LC gradient provided in the guidance document were chosen by 16 and 18 out the 23 participants, respectively (sometimes with a reduction of the final equilibration time). Various column temperatures (from 30 °C to 50 °C) and injection volumes (from 4 µL to 20 µL) were considered. All instruments operated in MS/MS mode using polarity switching, except three labs for which ESI was only used in positive mode. In this last case, ZEN was analyzed as its [M + H]^+^ adduct as already reported [22,23]. The diversity of the instrumentation (i.e., different generations, varying degrees of performance) used by participants was an additional proof of the applicability of the method.

To facilitate lab work, calibrant solutions (nine levels) were provided as ready-to-use to each participant. The extent of mycotoxin contaminations is known to be unpredictable and variable; thus, the calibration range of each analyte was set broad enough to avoid reinjection or re-analysis of highly contaminated samples. Participants were asked to consider the two highest levels (calibration point 7 (CAL 7) and CAL 8) only when facing such highly contaminated samples with concentrations out of the classical calibration range (CAL 0 to CAL 6). Use of a weighing factor (1/x or 1/x^2^) for drawing calibration curves was strongly recommended, or alternatively to force regression lines through the origin (i.e., intercept = 0), as done elsewhere [14], this to maintain good precision of data at low contamination levels. Typically, such an approach enabled the direct quantification of either AFB1 from 0.025 µg/kg to 32 µg/kg or OTA from 0.125 µg/kg to 32 µg/kg in cereals within one single analysis, avoiding a tedious re-extraction of the sample using a reduced test portion.

### 2.3. Laboratory Qualification

**Figure 2:**
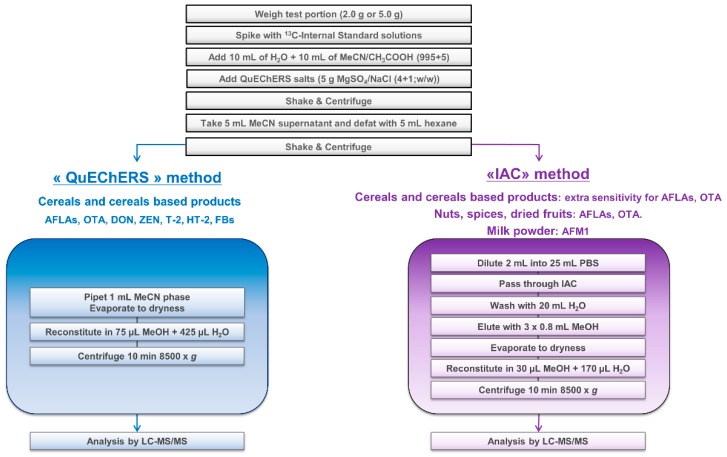
Overview of the sample preparation.

Participants were first asked to analyze one single sample (practice sample) to get familiar with the protocol and to communicate generated results to the study director. This was to ensure that the method was correctly set up before engaging laboratories in the second part of the study, consisting of the analysis of 28 samples. This practice sample being a maize based infant cereal, an IAC cleanup was required to get extra sensitivity for AFLAs and OTA. Other mycotoxins were extracted using the “QuEChERS” procedure (Figure 2). The 11 assigned values derived from the proficiency test were 0.26 µg/kg AFB1, 0.28 µg/kg AFB2, 0.15 µg/kg AFG1, 0.15 µg/kg AFG2, 0.81 µg/kg AFTOT, 0.71 µg/kg OTA, 138 µg/kg DON, 31 µg/kg ZEN, 31 µg/kg T-2, 27 µg/kg HT-2, 56 µg/kg T-2 + HT-2, 61 µg/kg FB1, 79 µg/kg FB2, and 140 µg/kg FBTOT, thus very close to the low regulated levels for foods intended for infants and young children [3]. Twenty (20) out of 23 participants successfully reported data with z-scores (Z) and recoveries (Rec) within −2 < Z < +2 and 70% < Rec < 130% for these 11 mycotoxins and were thus qualified for the second part of the study. At this stage, assistance provided by the study director was limited to a few participants facing issues in using the correct ISTD concentrations or eliminating FBs’ carry-over on their MS instrument. One laboratory did not see any peak for ZEN, OTA, FBs, HT-2, T-2, and DON, which are compounds to be detected by the QuEChERS stream. Investigations to identify the root cause failed. As the method could not be properly implemented in this lab, all data further generated were not considered for method performance evaluation. Another laboratory missed reporting practice sample data before starting the full study. Unfortunately, the z-scores for ZEN and AFG2 and recoveries for AFB1, AFB2 and AFG1 were unsatisfactory, which highlighted a lack of control of the procedure together with a possible instrumental issue. A third laboratory reported both technical and organizational problems and did not provide data for the practice sample. For consistency with other participants, the decision was made to remove these three laboratories for the final statistical evaluation. Consequently, 20 laboratories were eventually approved in the full collaborative study. 

### 2.4. Full Collaborative Study

Among the 3700 individual data tentatively collected, only a few of them were missing or excluded (*n* = 84, i.e., 2.3% of the data) before statistical evaluation for the following reasons: (a) one lab did not report DON and HT-2 toxin concentration data in the wheat based baby food due to ion ratios out of tolerance; (b) OTA level in the black pepper sample was discarded for one laboratory facing a low signal-to-noise-ratio (<3) along with a very bad peak shape; (c) one lab did not report data for ZEN in maize and wheat samples due to important baseline interference for ZEN ISTD; (d) all data for black pepper were removed for one lab because the sample preparation was not followed; (e) data for T-2 and HT-2 toxins were not considered for another laboratory reporting major instrumental issues for these two toxins; (f) an error during the ISTD spiking step (DON, ZEN, T-2, HT-2) of the wheat based infant cereal was mentioned by one laboratory.

**Figure 3:**
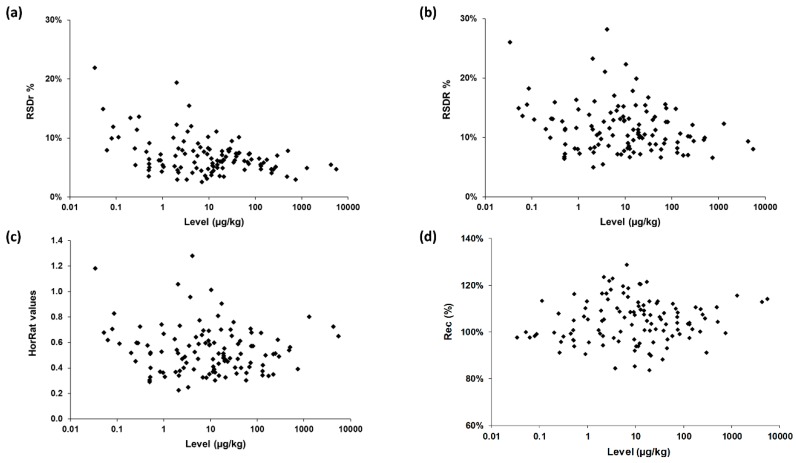
Method performances on 115 mycotoxin/sample combinations: (**a**) repeatability; (**b**) reproducibility; (**c**) Horwitz ratio (HorRat) values; (**d**) trueness. Rec, recovery.

The precision of the method was characterized by the repeatability RSD_r_ and the reproducibility RSD_R_, after removal of outliers as recommended in ISO 5725-2 [20]. As shown in Table 2, Table 3 and Table 4 and Figure 3, RSD_r_ ranged from 3% to 22% (average 7%) and RSD_R_ from 5% to 28% (average 12%), most values being largely below 20%. All these data fulfilled Commission Regulation (EC) No. 401/2006 [24], which has established method performance criteria for the official control of the levels of mycotoxins in foodstuffs. As expected, reproducibility values were higher than the repeatability ones for all combinations except three. 

The Horwitz ratio (HorRat) is a useful index of method performance with respect to precision and is calculated as the ratio between the RSD_R_ as obtained during the collaborative study and the RSD_R_ as predicted by the modified Horwitz equation [25,26]. Method reproducibility is considered as normal when the HorRat value is between 0.5 and 1.5 [19]. In this study, HorRat values ranged from 0.2 to 1.3 for the 115 mycotoxin/sample combinations (Table 2, Table 3 and Table 4, Figure 3). Approximately 50% of HorRat values were even lower than 0.5. This might be explained by: (a) the high robustness of the method (already known since it has been heavily used for >5 years in routine environments at Nestlé), (b) by the use of isotopically labelled internal standards for quantification purpose, and (c) by the analytical skills of participating laboratories. 

The trueness of the method was then assessed by calculating the recovery (Rec, %) for each of the 115 mycotoxin/matrix combinations. Rec figures ranged from 84% to 129% with eleven data slightly above the requirements defined in EC 401/2006 [24]. We mention that three of them concerned FB1, FB2, and FBs in the maize sample for which levels were out of the calibration range (data reported for information purposes only). The overall set of data unambiguously demonstrated the high confidence level of the method regardless of the sample/concentration/mycotoxin combinations.

Focusing on individual participant data, all mycotoxins were correctly detected of the sample except in two cases. While all laboratories detected AFG2 at 0.31 µg/kg in the wheat based infant cereal, five of them could not detect AFG2 at a lower level in the rice based infant cereal (0.052 µg/kg), which could jeopardize the conclusion with respect to method sensitivity. However, neither AFG2 nor AFTOT (sum of the four AFLAs) are regulated for infant cereals. AFM1 in each of the two milk powders was detected by 16 and 20 out of the 20 laboratories, respectively. According to the related supplier recommendation, respective concentration levels in reconstituted milk correspond to 0.0034 µg/kg and 0.0117 µg/kg. Consequently, the highest AFM1 validated level that was detected by all participants was still half of the ML set for AFM1 in milk-based products (0.025 µg/kg [3]). 

As shown in Table 5, the lowest level of the sum of T-2 and HT-2 validated in this study was 18.6 µg/kg (i.e., 10.6 µg/kg and 8.0 µg/kg for T-2 and HT-2 as individual toxin, respectively). This is slightly higher than the 15 µg/kg level set for cereal based foods for infants and young children as an indicative level for which subsequent investigations should be performed according to EC 165/2013 [4]. However, internal validation data at 10 µg/kg for the sum of T-2 and HT-2 (i.e., 5 µg/kg for each T-2 and HT-2 toxins) [14,16] demonstrated the good performance of this method and its compliance with EC 165/2013 [4]. For all other mycotoxins considered, the lowest validated level in this study was lower than the lowest EU MLs [3], evidencing the method as fit for purpose to ensure compliance of food materials with regards to this regulation. 

### 2.5. Output: Future Perspective

Whilst LC-MS/MS based methods are recognized as state-of-the-art approaches for chemical contaminants’ analysis in food, current standards for mycotoxin monitoring are still mainly based on TLC or HPLC-UV/FLD and/or often limited to a single compound, or family at best [13]. This study proved that the proposed methodology is an excellent candidate for future standardization since it offers a highly trustful and efficient approach for multi-residue analysis of regulated mycotoxins. Its applicability whatever food type, mycotoxin, and concentration level is well adapted for food control environments, today facing shorter and shorter turn-around times. 

The cost effectiveness of any routine method is not to be neglected, and the price of ^13^C-labeled compounds used as ISTD might impede their usage. However, our own estimation showed that the sample throughput by using this isotopic dilution approach was increased by a factor of three, which was balanced with the additional cost of the ISs. The matrix matched calibration curve (which is restricted to one or two sample types analyzed per batch with the additional requirement to have blank matrices available for spiking purposes) or standard addition (requiring several extractions for one single analysis) quantifications are indeed time consuming, not user friendly, and not adapted for laboratories dealing with a broad range of food matrices on a daily basis. The use of labelled ISTD also improves method precision and accuracy, as recently highlighted in a study evaluating different approaches (extraction, clean-up, quantification) for mycotoxins determination in cereals [27]. The best performances were achieved by a method making use of ^13^C-labeled internal standards for quantification. 

Finally, the proposed extraction procedure (QuEChERS) is generic and already known to be efficient for a wide range of compounds with different polarity. Our trials already demonstrated that such an extraction approach is valid as well for deoxynivalenol acetylated or modified forms (e.g., 3-acetyl-DON, 15-acetyl-DON, DON-3-glucoside). These compounds are not regulated yet, but the European Commission initiated discussions to review the existing maximum levels for DON, taking into account DON acetylated and modified forms [28]. Related isotopically labelled compounds are now commercially available, making these compounds good candidates for future scope extension.

## 3. Conclusions

Standardized and internationally accepted methods for food compliance testing are of utmost importance to avoid discrepancies in results, as this may generate unsubstantiated disputes and ultimately distort global food trade. The current standardized methodologies devoted to the analysis of mycotoxins in food are not adapted anymore to high-throughput routine environments, facing today a broader range of items to monitor, an increasing pressure to shorten turn-around time, and cost constraints. This paper proposes an efficient analytical approach for multi-residue analysis of regulated mycotoxins, even at low regulated levels for foods intended for infants and young children. The overall set of data derived from this study proved that the proposed methodology is an excellent candidate for future standardization.

## 4. Materials and Methods

### 4.1. Study Organization

#### 4.1.1. Study Materials

Each participant was supplied with AFLAOCHRA PREP^®^ IACs (R-Biopharm, Darmstadt, Germany, *n* = 26), ready-to-use QuEChERS salt mixtures (Agilent, Geneva, Switzerland, *n* = 35), and ready-to-use PBS tablets (Oxoid, Basingstoke, U.K., *n* = 10). 

#### 4.1.2. Study Samples

Each participant was given 30 bottles containing ca. 25.0 g of foods. Samples were composed of 28 blind duplicate samples randomly coded from A to β and of one “practice sample” in duplicate. Samples A to L (*n* = 12) belonged to the group “nuts, spices, and dried fruits” and were paprika, black pepper, almonds, hazelnuts, dried raisins, and dried figs. Samples M to R were “raw cereals” composed of wheat or maize (*n* = 6). Samples S to X were either rice, wheat, or maize based “infant cereals” (*n* = 6). Samples Y to β were “milk powder” (*n* = 4). The “practice sample” was a maize based infant cereal. All samples were in powdered form except hazelnuts and dried raisins, which were provided as slurry paste. 

These samples were prepared by Fapas (York, U.K.) or BIPEA (Paris, France). All items but one were former proficiency test materials from the last 2 years, still commercially available as QC materials. They were initially packaged in 200-g aluminum bags, but further re-dispatched in smaller 25-g units after extensive mixing. Mycotoxins were either naturally present in the material and/or spiked by the supplier to reach the level of interest. Only the rice based infant cereal sample was specifically prepared by BIPEA using a mycotoxin-free product available from a supermarket in France. The item was further spiked at levels close to the first calibration point (i.e., at the limit of quantification).

#### 4.1.3. Study Analytical Standards Solutions for Spiking Purposes

Five ready-to-use ^13^C-labelled mycotoxin mixtures to be used as internal standards were prepared by Romer Labs (Tulln, Austria): (a) the ^13^C-AFLA mixture was composed of (^13^C_17_)-AFB1, (^13^C_17_)-AFB2, (^13^C_17_)-AFG1, and (^13^C_17_)-AFG2, each at 0.5 µg/mL in acetonitrile; (b) the ^13^C-AFM1 solution was composed of (^13^C_17_)-AFM1 at 0.1 µg/mL in acetonitrile; (c) the ^13^C-[DON, T-2, HT-2, ZEN] mixture was composed of (^13^C_15_)-DON, (^13^C_24_)-T-2 toxin, (^13^C_22_)-HT-2 toxin, and (^13^C_18_)-ZEN, at 5, 2.5, 2.5, and 2 µg/mL in acetonitrile, respectively; (d) the ^13^C-FBs solution was composed of (^13^C_34_)-FB1 and (^13^C_34_)-FB2, each at 10 µg/mL in acetonitrile/water (50 + 50); (e) ^13^C-OTA was composed of (^13^C_20_)-OTA at 10 µg/mL in acetonitrile. 

These prepared solutions were dispatched as such to all participants with the exception of ^13^C-OTA, which was subsequently diluted at 0.1 µg/mL in methanol-water (85 + 15) by the study organizer.

#### 4.1.4. Study Analytical Standard Solutions for External Calibration Curves

Unlabeled mycotoxin working standard solutions were provided by Romer Labs and were: (a) the AFLA mixture composed of AFB1, AFB2, AFG1, and AFG2, each at 1 µg/mL in acetonitrile; (b) AFM1 at 0.1 µg/mL in acetonitrile; (c) the DON, T-2, HT-2, and ZEN mixture at 5, 2.5, 2.5, and 2 µg/mL in acetonitrile, respectively; (d) the FB mixture composed of FB1 and FB2, each at 5 µg/mL in acetonitrile-water (50 + 50); (e) OTA at 10 µg/mL in acetonitrile. The AFLA mixture was then diluted at 0.1 µg/mL and 0.01 µg/mL in acetonitrile and OTA at 0.1 µg/mL in methanol-water (15 + 85).

To ease lab work, a set of nine individual “ready-to-be injected” calibration points (named CAL 0 to CAL 8) was also provided. Related concentrations are given in the Supplementary Data. The same batch of each labelled mycotoxin mixture was intended to be used for both making calibration solutions and spiking test portions. These ready-to-use calibration solutions were all prepared at Nestlé Research Lausanne (Lausanne, Switzerland). A total of 30 mL of each of individual calibration solution was prepared and aliquoted in crimp vials before being stored at −20 °C. A previous internal stability study evidenced that such calibration solutions are stable for at least 4 months when stored at −20 °C.

Before use, all calibration solutions were brought to room temperature, extensively vortexed (minimum 1 min), and sonicated (approximately 10 min) to ensure efficient (re)solubilization of all analytes. 

#### 4.1.5. Shipping Study Materials

Initially, 26 laboratories enrolled in this study, but two labs could not receive materials due to customs issues (Romania, Russia). One lab withdrew its participation due to a lack of available time. Thus, 23 packages were finally dispatched to laboratories in Austria, France, Germany (2), Hungary, India, Ireland (2), Italia (5), Poland, Serbia, Singapore, Switzerland, The Netherlands (2), The United Kingdom (2), and the USA (2) in June 2019.

Samples and mycotoxin standards’ solutions were all sent in a frozen state (dry ice), whereas other materials were sent at room temperature.

### 4.2. Sample Preparation

Participants were asked to follow strictly the provided protocol. Samples were extracted as previously reported [14] with minor modifications. 

Test portions (5 g for cereals and cereal based products and 2 g for milk powders, nuts, spices, and dried fruits) were weighed in 50 mL polypropylene tubes and each subsequently spiked with 50 µL of ^13^C-labelled working standard solutions of interest. Water (10 mL) was added, and the tube was vigorously shaken by hand until complete dissolution. Acetonitrile containing 1% acetic acid (10 mL) was added, and the tube was then mechanically shaken for 10 min at approximately 300 rpm. Ready-to-use QuEChERS salt mixture containing 4.0 ± 0.1 g of MgSO_4_ and 1.0 ± 0.1 g of NaCl was supplemented to initiate phase separation. The tube was immediately hand shaken to prevent any lump formation and then placed onto a mechanical shaker for 10 min. After centrifugation (4000× *g*, 10 min, room temperature (RT)), 5 mL of the supernatant acetonitrile phase were mixed with 5 mL of n-hexane and shaken for approximately 10 min on a mechanical shaker. After centrifugation (4000× *g*, 1 min, RT), the upper n-hexane phase was discarded. 

The sample extract was then divided into two portions and submitted to two different clean-up protocols, named “QuEChERS” and “IAC”, depending on the mycotoxin/matrix combination and the sensitivity required for AFLAs and OTA. A general scheme is presented in Figure 1:

- “QuEChERS”: Generic cleanup for all mycotoxins potentially present in cereals when an improved sensitivity for AFLAs and OTA is not required. An aliquot of the defatted acetonitrile layer (1 mL) was evaporated to dryness under a stream of nitrogen at about 40 °C and reconstituted in 75 µL methanol and 425 µL water. The resulting extract was mixed for about 5 s using a vortex mixer and ultracentrifuged at 8500× *g* at room temperature for 10 min.
- “IAC”: Specific cleanup for AFLAs and OTA for sensitivity purposes when dealing with infant foods (e.g., infant cereals) and “difficult” matrices (e.g., spices, nuts, dried fruits). An aliquot of the acetonitrile layer (2 mL) was diluted in a PBS solution (25 mL), and the whole extract was applied onto IAC containing antibodies specific to AFLAs and OTA. The IAC was then washed with 20 mL water and the toxins finally eluted with methanol (3 × 800 µL). The eluate was evaporated to dryness under a stream of nitrogen at about 40 °C and reconstituted in 30 µL of methanol and 170 µL of water. The resulting extract was mixed for about 5 s using a vortex mixer and ultracentrifuged at 8500× *g* at room temperature for 10 min.

### 4.3. LC-MS/MS Analysis

Instrumental conditions described hereafter were given to the participants, who were free to adapt them for their own instrument. 

LC analysis was performed with an Agilent 1290 binary pump system (Agilent, Geneva, Switzerland). Optimal LC conditions were obtained using a Waters Acquity BEH C18 column (2.1 × 100 mm, 1.7 µm) equipped with a BEH C18 VanGuard precolumn (2.1 × 5 mm, 1.7 μm), both thermostated at 50 °C. The mobile phases were constituted of formic acid (0.15%) in water containing 10 mM of ammonium formate (Solvent A) and formic acid (0.05%) in methanol (Solvent B). A gradient program was set up as follows: 0–0.3 min with 85% A; 0.3–4.0 min linear gradient down to 0% A; hold at 0% A for 3 min; return to 85% A in 0.05 min and hold at 85% A for 3.95 min (total run time 10 min). The flow rate was 0.4 mL/min, and the injection volume was 10 µL. The LC flow was directed into the MS detector between 1.0 and 6.0 min. 

MS detection was performed using a Sciex TRIPLE QUAD 6500+ instrument (Foster City, USA) equipped with a Turbo V™ ion source. MS parameters were first obtained by syringe-infusing (each individual standard solution in electrospray ionization (ESI) mode at concentrations of ca. 0.1 µg/mL to 1.0 µg/mL along with the LC flow (0.4 mL/min, constituted of 50% Aqueous Mobile Phase A and 50% Organic Mobile Phase B) using a T connector. Analyses were conducted using tandem MS in scheduled selected reaction monitoring (scheduled MRM^TM^) mode alternating two transition reactions for each compound with an acquisition window of 40 s and a target scan time of 250 ms. The block source temperature was maintained at 550 °C, and gas values were set as follows: curtain gas 35 psi, GS1 40 psi, GS2 40 psi, and collision activated dissociation (CAD) gas at 10 psi. Other parameters were ion spray voltage (5.0 kV or −4.0 kV), entrance potential (±10 eV), and collision exit potential (±15 eV). MS/MS operated in positive/negative ionization switching mode. Individual MS parameters for the 24 compounds are provided in the Supplementary Data. Data acquisition was carried out using Analyst software 1.7 and subsequent data processing done using Multiquant software 3.0 (both from Sciex). 

### 4.4. Identification of Mycotoxins 

Mycotoxins were considered as positively identified in the sample when all confirmation criteria defined in the SANTE/12089/2016 document [29] were fulfilled: (a) a signal visible at least at two diagnostic transition reactions selected for each mycotoxin and each corresponding IS; (b) the retention time of the analyte in the sample extract corresponds to that of the average of the calibration standards measured in the same sequence with a tolerance of ±0.2 min; (c) the retention time of the analyte corresponds to that of its labelled internal standard with a tolerance of ±0.05 min; (d) the peak area ratio from the different transition reactions recorded for each analyte is ±30%.

### 4.5. Quantification

Quantification was performed by the isotopic dilution approach using ^13^C-labeled mycotoxins as internal standard. For each mycotoxin, the calibration curve was built by plotting the peak area ratio of each mycotoxin and its ISTD using the transition reaction for quantitation (= *y* axis) against the concentration ratio of each mycotoxin and its ISTD (= *x* axis) using calibration solutions from CAL 0 to CAL 6. CAL 7 and CAL 8 were only considered in the case of a highly contaminated sample when mycotoxin levels were out of the classical calibration range. 

To improve the precision of the results at the low calibration points, participants were encouraged to use a 1/x or 1/x^2^ weighing factor for drawing calibration curves. Alternatively, the regression line was forced through the origin (i.e., intercept = 0) as initially validated [14]. Deviations of back calculated concentrations of calibrant standards from the true concentration were checked to be below ±20% [30].

The mass fraction of each analyte in the sample (*w_a_*) in µg/kg was calculated using the following equation:

(1) $$W_{a}\;=\frac{\left(\frac{A_{a}}{A_{is}}\right)-I}{S}\times \frac{m_{is}}{m_{a}}$$

where *A_a_* is the peak area of a given analyte; *A_is_* is the peak area of the corresponding ISTD; *I* is the intercept of the regression line; *S* is the slope of the regression line; ma is the mass of the test portion, in g (either 2.0 g or 5.0 g); *m_is_* is the mass of *ISTD* added to the test portion, in ng.

### 4.6. Statistical Evaluation

Each participating laboratory was first asked to analyze a “practice sample” by strictly following the protocol given and to report results to the study director. Such a preliminary trial was intended to familiarize the participant with the procedure and to ensure that the method was correctly set up before starting the full study. The practice sample was an old proficiency test sample. Therefore, for each laboratory, z-scores and recoveries were calculated based on the *p*-test assigned values (BIPEA, Babyfood, round 12-3931, December 2018). When z-scores (Z) and recoveries (Rec) were within −2 < Z < +2 and 70% < Rec < 130% ranges, the laboratory was qualified for the second part of the study. When a laboratory did not return satisfactory results, technical support was provided to fix issues.

Method performances were then assessed based on ISO 5725-2 [20] and AOAC International [19] procedures using the data from the 28 blind duplicate samples that were analyzed over a maximum of four different days by the qualified labs. After data collection, outliers and stragglers were detected using Cochran and Grubbs tests (with critical values set at 1% and 5%, respectively). Outliers were removed prior to calculations, but stragglers were retained. Statistics for AFTOT, FBTOT, and T2 + HT-2 toxins were derived from the sum of individual mycotoxin concentrations. Averaged concentrations, standard deviations of repeatability (SDr), and relative standard deviations of repeatability (RSD_r_) were estimated from blind duplicates for each sample and each mycotoxin. Standard deviations of reproducibility (SDR), relative standard deviations of reproducibility (RSD_R_), and HorRat values were also calculated. Trueness was evaluated based on the assigned values given by proficiency test manufacturers for each individual material, except for Sample S12 (Table 2 and Table 3) for which reference values were obtained using our internal method (six replicates).

## Figures and Tables

**Table 1:** Official methods (*n* = 72) for the determination of mycotoxins in food (AOAC, CEN, ISO). ELISA, enzyme-linked immunosorbent assay; HPLC-FLD, high performance liquid chromatography with fluorescence detection; IAC, immunoaffinity column; TLC, thin layer chromatography; HPLC-UV, high performance liquid chromatography with ultra violet detection; GC, gas chromatography.

Mycotoxin(s)	Document	Scope	Technique
AFB1	AOAC 990.32	Corn, roasted peanuts	ELISA
AFB1	AOAC 2000.16	Infant formula	HPLC-FLD (IAC)
AFB1	EN 15851:2010	Cereals, cereal based foods ^1^	HPLC-FLD (IAC)
AFB1	AOAC 978.15	Egg	TLC
AFB1, AFB2, AFG1, AFG2	AOAC 990.34	Corn, cotton seed, peanuts, peanut butter	ELISA
AFB1, AFB2, AFG1, AFG2	AOAC 991.31	Corn, raw peanuts, peanut butter	HPLC-FLD (IAC)
AFB1, AFB2, AFG1, AFG2	AOAC 2005.08	Corn, raw peanuts, peanut butter	HPLC-FLD (IAC)
AFB1, AFB2, AFG1, AFG2	AOAC 994.08	Corn, almonds, nuts, peanuts, pistachio nuts	HPLC-FLD
AFB1, AFB2, AFG1, AFG2	AOAC 968.22	Peanuts and peanut products	TLC
AFB1, AFB2, AFG1, AFG2	AOAC 970.45	Peanuts and peanut products	TLC
AFB1, AFB2, AFG1, AFG2	AOAC 972.27	Soybeans	TLC
AFB1, AFB2, AFG1, AFG2	AOAC 971.23	Cocoa beans	TLC
AFB1, AFB2, AFG1, AFG2	AOAC 971.24	Coconut, copra	TLC
AFB1, AFB2, AFG1, AFG2	AOAC 972.26	Corn	TLC
AFB1, AFB2, AFG1, AFG2	AOAC 993.17	Corn, peanuts	TLC
AFB1, AFB2, AFG1, AFG2	AOAC 970.46	Green coffee	TLC
AFB1, AFB2, AFG1, AFG2	AOAC 974.16	Pistachio nuts	TLC
AFB1, AFB2, AFG1, AFG2	AOAC 998.03	Shelled peanuts	TLC
AFB1, Total AFLAs	AOAC 990.33	Corn, peanut butter	HPLC-FLD
AFB1, Total AFLAs	ISO 16050:2003	Cereals, nuts, oilseed products, dried fruits	HPLC-FLD
AFB1, Total AFLAs	AOAC 999.07	Peanut butter, pistachio paste, fig paste, paprika	HPLC-FLD (IAC)
AFB1, Total AFLAs	EN 14123:2007	Hazelnuts, peanuts, pistachios, figs, paprika	HPLC-FLD (IAC)
Total AFLAs	AOAC 991.45	Peanut butter	ELISA
Total AFLAs	AOAC 993.16	Corn	ELISA
Total AFLAs	AOAC 2013.05	Olive oil, peanut oil, sesame oil	HPLC-FLD (IAC)
Total AFLAs	AOAC 975.36	Corn, peanut, peanut butter, pistachio nuts	UV Lamp
Total AFLAs	AOAC 979.18	Corn, raw and shelled peanuts	Visual fluorescence
AFM1	ISO 14675:2003	Milk products	ELISA
AFM1	ISO 14501:2007	Milk products	HPLC-FLD
AFM1	AOAC 2000.08	Liquid milk	HPLC-FLD (IAC)
AFM1	ISO 14674:2005	Milk products	TLC
AFM1	AOAC 974.17	Dairy products	TLC
AFM1	AOAC 980.21	Milk, cheese	TLC
AFM1	AOAC 982.26	Liver	TLC
AFM1, AFB1	AOAC 982.24	Liver	TLC
AFM1, AFB1	AOAC 982.25	Liver	TLC
AFM1, AFM2 ^2^	AOAC 986.16	Liquid milk	HPLC-FLD
CIT ^3^	EN 17203:2018	Cereals, red yeast rice	LC-MS/MS
DON	AOAC 986.18	Wheat	GC
DON	EN 15891:2010	Cereals, cereal based foods ^1^	HPLC-UV
DON	AOAC 986.17	Wheat	TLC
FB1, FB2	AOAC 2001.04	Corn, corn flakes	HPLC-FLD (IAC)
FB1, FB2	EN 16187:2015	Maize based food ^1^	HPLC-FLD (IAC)
FB1, FB2	EN 14352:2004	Maize based food	HPLC-FLD (IAC)
FB1, FB2, FB3 ^4^	AOAC 995.15	Corn	HPLC-FLD
Total FBs	AOAC 2001.06	Corn	ELISA
OTA	AOAC 991.44	Barley, wheat, rye, corn	HPLC-FLD
OTA	ISO 15141:2018	Cereals	HPLC-FLD
OTA	AOAC 2000.03	Barley	HPLC-FLD (IAC)
OTA	AOAC 2000.09	Roasted coffee	HPLC-FLD (IAC)
OTA	AOAC 2001.01	Wines, beer	HPLC-FLD (IAC)
OTA	AOAC 2004.10	Green coffee	HPLC-FLD (IAC)
OTA	EN 15835:2010	Cereal based food	HPLC-FLD (IAC)
OTA	EN 15829:2010	Dried fruits (currants, raisins, sultanas, figs)	HPLC-FLD (IAC)
OTA	EN 14133:2009	Wine, beer	HPLC-FLD (IAC)
OTA	EN 14132:2009	Barley, roasted coffee	HPLC-FLD (IAC)
OTA	ISO 15141:2018	Cereals, cereal based products	HPLC-FLD (IAC)
OTA	AOAC 975.38	Green coffee	TLC
OTA, OTB ^5^	AOAC 973.37	Barley	TLC
Patulin	ISO 8128-2:1993	Apple juice and apple juice based products	HPLC-UV
Patulin	EN 15890:2010	Fruit juice and fruit based purée ^1^	HPLC-UV
Patulin	EN 14177:2003	Clear and cloudy apple juice and puree	HPLC-UV
Patulin	AOAC 995.10	Apple juice	HPLC-UV
Patulin	AOAC 2000.02	Apple juice, apple puree	HPLC-UV
Patulin	ISO 8128-1:1993	Apple juice and apple juice based products	TLC
Patulin	AOAC 974.18	Apple juice	TLC
T-2, HT-2	EN 16923:2017	Cereals, cereal based foods ^1^	LC-MS/MS
ZEN	AOAC 994.01	Corn, wheat, feed	ELISA
ZEN	EN 15850:2010	Cereals, cereal based foods ^1^	HPLC-FLD (IAC)
ZEN	EN 16924:2017	Edible vegetable oils	HPLC-FLD or
			LC-MS/MS
ZEN	AOAC 976.22	Corn	TLC
ZEN, α-ZEL ^6^	AOAC 985.18	Corn	HPLC-FLD

^1^ Including foods intended for infants and young children; ^2^ aflatoxin M2; ^3^ citrinin; ^4^ fumonisin B3; ^5^ ochratoxin B; ^6^ α-zearalenol.

**Table 2:** Method performance evaluation for AFB1, AFB2, AFG1, AFG2, Total AFLAs and OTA in twelve samples.

		S1	S2	S3	S4	S5	S6	S7	S8	S9	S10	S11	S12
**AFB1**	Assigned Value (µg/kg)	5.4	11.4	11.3	6.54	8.2	2.48	4.86	2	4.58	0.26	0.51	0.0857
	No. of Laboratories	20	20	20	19	20	20	20	20	20	20	20	19
	No. of Outliers	1	2	2	0	2	1	2	0	0	0	2	0
	No. of Accepted Results	19	18	18	19	18	19	18	20	20	20	18	19
	Mean (µg/kg)	6.46	12.7	12.7	8.42	8.44	2.89	4.86	2.33	4.97	0.237	0.491	0.0848
	SDr (µg/kg)	0.32	0.53	0.47	0.71	0.3	0.14	0.29	0.45	0.39	0.013	0.028	0.0101
	RSDr (%)	5	4	4	8	4	5	6	19	8	5	6	12
	SDR (µg/kg)	0.67	1.15	1.31	1.22	0.6	0.3	0.69	0.54	0.57	0.031	0.036	0.0155
	RSDR (%)	10	9	10	15	7	10	14	23	12	13	7	18
	Rec (%)	120	111	113	129	103	116	100	116	109	91	96	99
	HorRat Values	0.5	0.4	0.5	0.7	0.3	0.5	0.6	1.1	0.5	0.6	0.3	0.8
**AFB2**	Assigned Value (µg/kg)	3.3	12.5	0.9	5.59	4.4	0.96	n.d.	1.9	2.01	0.25	0.5	0.0792
	No. of Laboratories	20	20	20	19	20	20	-	18	20	20	20	19
	No. of Outliers	2	2	0	2	1	1	-	1	0	0	1	2
	No. of Accepted Results	18	18	20	17	19	19	-	17	20	20	19	17
	Mean (µg/kg)	4.06	13.7	0.991	6.52	4.22	1.09	-	2.07	2.1	0.269	0.483	0.0778
	SDr (µg/kg)	0.12	0.71	0.062	0.46	0.17	0.05	-	0.15	0.26	0.022	0.022	0.0077
	RSDr (%)	3	5	6	7	4	4	-	7	12	8	5	10
	SDR (µg/kg)	0.22	0.91	0.115	0.84	0.36	0.09	-	0.25	0.24	0.027	0.031	0.0121
	RSDR (%)	5	7	12	13	9	8	-	12	11	10	6	16
	Rec (%)	123	109	110	117	96	113	-	109	105	108	97	98
	HorRat Values	0.2	0.3	0.5	0.6	0.4	0.4	-	0.5	0.5	0.5	0.3	0.7
**AFG1**	Assigned Value (µg/kg)	2.1	20.9	9.5	3.03	1.8	2.65	n.d.	1.7	7.77	0.2	0.5	0.0628
	No. of Laboratories	20	20	20	19	20	20	-	18	20	20	20	19
	No. of Outliers	1	1	1	2	0	0	-	1	0	1	0	3
	No. of Accepted Results	19	19	19	17	20	20	-	17	20	19	20	16
	Mean (µg/kg)	2.06	21.6	10.2	3.58	1.79	3.02	-	1.71	8.42	0.199	0.504	0.0613
	SDr (µg/kg)	0.094	0.75	0.49	0.26	0.093	0.24	-	0.17	0.65	0.027	0.018	0.0049
	RSDr (%)	5	3	5	7	5	8	-	10	8	13	4	8
	SDR (µg/kg)	0.15	1.55	0.79	0.35	0.15	0.27	-	0.24	1.1	0.023	0.034	0.0083
	RSDR (%)	7	7	8	10	8	9	-	14	13	11	7	14
	Rec (%)	98	103	107	118	99	114	-	101	108	100	101	98
	HorRat Values	0.3	0.3	0.4	0.4	0.4	0.4	-	0.6	0.6	0.5	0.3	0.6
**AFG2**	Assigned Value (µg/kg)	2.2	15	11.9	2.84	0.9	0.83	n.d.	3.7	5.79	0.31	0.52	0.052
	No. of Laboratories	20	20	20	19	19	19	-	19	20	20	20	15
	No. of Outliers	2	1	0	2	1	3	-	0	0	1	2	1
	No. of Accepted Results	18	19	20	17	18	16	-	19	20	19	18	14
	Mean (µg/kg)	2.32	16.4	11.3	3.46	0.814	0.885	-	3.13	6.14	0.304	0.488	0.052
	SDr (µg/kg)	0.1	1.01	0.65	0.33	0.059	0.055	-	0.48	0.56	0.041	0.045	0.0077
	RSDr (%)	4	6	6	9	7	6	-	15	9	14	9	15
	SDR (µg/kg)	0.19	1.85	0.97	0.37	0.133	0.072	-	0.66	1.05	0.048	0.043	0.0077
	RSDR (%)	8	11	9	11	16	8	-	21	17	16	9	15
	Rec (%)	105	109	95	122	90	107	-	84	106	98	94	100
	HorRat Values	0.4	0.5	0.4	0.5	0.7	0.4	-	1	0.8	0.7	0.4	0.7
**Total AFLAs**	Assigned Value (µg/kg)	12.5	58.4	33.8	19.2	15.2	6.81	4.86	9.2	20.2	1.05	2.07	0.28
	No. of Laboratories	20	20	20	19	20	19	20	20	20	20	20	19
	No. of Outliers	0	0	0	0	0	1	2	0	0	1	1	1
	No. of Accepted Results	20	20	20	19	20	18	18	20	20	19	19	18
	Mean (µg/kg)	15	65.4	35.6	21.7	15.3	7.83	4.86	8.9	21.6	1	1.95	0.268
	SDr (µg/kg)	0.67	3.04	2.09	1.3	0.76	0.35	0.29	0.65	1.36	0.051	0.058	0.0305
	RSDr (%)	4	5	6	6	5	4	6	7	6	5	3	11
	SDR (µg/kg)	1.22	5.22	3.16	2.36	1.15	0.56	0.69	1.36	2.16	0.073	0.097	0.0351
	RSDR (%)	8	8	9	11	8	7	14	15	10	7	5	13
	Rec (%)	120	112	105	113	101	115	100	97	107	95	94	96
	HorRat Values	0.4	0.4	0.4	0.5	0.3	0.3	0.6	0.7	0.5	0.3	0.2	0.6
**OTA**	Assigned Value (µg/kg)	16.9	7	12	17.2	1	9.05	3.46	4.1	2.2	0.52	0.51	0.448
	No. of Laboratories	20	20	20	18	20	20	20	20	20	20	20	20
	No. of Outliers	1	0	0	1	0	1	2	0	2	1	0	0
	No. of Accepted Results	19	20	20	17	20	19	18	20	18	19	20	20
	Mean (µg/kg)	17.7	8.3	14.5	20.9	1.05	9.81	3.37	4.51	2.72	0.6	0.535	0.444
	SDr (µg/kg)	0.64	0.21	0.77	1.26	0.06	0.31	0.37	0.54	0.22	0.03	0.0345	0.0341
	RSDr (%)	4	3	5	6	5	3	11	12	8	5	6	8
	SDR (µg/kg)	2.73	1.27	1.91	4.16	0.16	1.33	0.43	1.27	0.44	0.069	0.06	0.056
	RSDR (%)	15	15	13	20	15	14	13	28	16	11	11	13
	Rec (%)	105	119	121	122	105	108	97	110	123	116	105	99
	HorRat Values	0.7	0.7	0.6	0.9	0.7	0.6	0.6	1.3	0.7	0.5	0.5	0.6

S1: paprika; S2: hazelnuts, S3: dried raisins; S4: black pepper; S5: almond powder; S6: Figs; S7: maize; S8: maize; S9: wheat; S10: wheat based infant cereal; S11: maize based infant cereal; S12: rice based infant cereal; SDr: standard deviations of repeatability; RSDr: relative standard deviations of repeatability; SDR: standard deviations of reproducibility; RSDR: relative standard deviations of reproducibility; Rec: recovery; n.d.: not detected.

**Table 3:** Method performance evaluation for ZEN, DON, T-2, HT-2, T-2+HT-2, FB1, FB2, Total FBs in six samples.

		S7	S8	S9	S10	S11	S12
**ZEN**	Assigned Value (µg/kg)	5.4	11.4	11.3	6.54	8.2	2.48
	No. of Laboratories	20	20	20	19	20	20
	No. of Outliers	1	2	2	0	2	1
	No. of Accepted Results	19	18	18	19	18	19
	Mean (µg/kg)	6.46	12.7	12.7	8.42	8.44	2.89
	SDr (µg/kg)	0.32	0.53	0.47	0.71	0.3	0.14
	RSDr (%)	5	4	4	8	4	5
	SDR (µg/kg)	0.67	1.15	1.31	1.22	0.6	0.3
	RSDR (%)	10	9	10	15	7	10
	Rec (%)	120	111	113	129	103	116
	HorRat Values	0.5	0.4	0.5	0.7	0.3	0.5
**DON**	Assigned Value (µg/kg)	743	506	176	220	295	45.2
	No. of Laboratories	20	20	20	18	20	20
	No. of Outliers	1	0	1	1	0	0
	No. of Accepted Results	19	20	19	17	20	20
	Mean (µg/kg)	739	527	195	220	269	43.7
	SDr (µg/kg)	22	41.5	12.3	10.4	18.9	3.3
	RSDr (%)	3	8	6	5	7	8
	SDR (µg/kg)	48.5	52.5	13.6	15.5	25.3	3.86
	RSDR (%)	7	10	7	7	9	9
	Rec (%)	99	104	111	100	91	97
	HorRat Values	0.4	0.6	0.3	0.3	0.5	0.4
**T-2**	Assigned Value (µg/kg)	57.9	43	10.3	15	23.3	11.3
	No. of Laboratories	19	19	19	18	19	19
	No. of Outliers	1	0	0	0	0	2
	No. of Accepted Results	18	19	19	18	19	17
	Mean (µg/kg)	56.9	44.4	9.66	16.1	22.2	10.6
	SDr (µg/kg)	3.5	3.29	0.78	0.89	1.52	0.7
	RSDr (%)	6	7	8	6	7	7
	SDR (µg/kg)	3.78	5.98	2.15	1.54	2.2	0.87
	RSDR (%)	7	13	22	10	10	8
	Rec (%)	98	103	94	107	95	94
	HorRat Values	0.3	0.6	1	0.4	0.5	0.4
**HT-2**	Assigned Value (µg/kg)	81.8	27	28.6	19	14.5	9.5
	No. of Laboratories	19	19	19	17	19	19
	No. of Outliers	0	0	0	1	0	0
	No. of Accepted Results	19	19	19	16	19	19
	Mean (µg/kg)	81.1	30.3	32.3	15.9	16.1	8.1
	SDr (µg/kg)	5.19	2.34	2.65	1.24	1.79	0.82
	RSDr (%)	6	8	8	8	11	10
	SDR (µg/kg)	10.2	4.67	4.65	1.8	2.88	1.03
	RSDR (%)	13	15	14	11	18	13
	Rec (%)	99	112	113	83	111	85
	HorRat Values	0.6	0.7	0.7	0.5	0.8	0.6
**T-2 + HT-2**	Assigned Value (µg/kg)	132	68	38.9	36	38.2	20.8
	No. of Laboratories	19	19	19	17	19	19
	No. of Outliers	0	0	0	1	0	0
	No. of Accepted Results	19	19	19	16	19	19
	Mean (µg/kg)	137	74.7	42	31.8	38.4	18.7
	SDr (µg/kg)	6.49	4.43	2.89	1.98	2.83	1.17
	RSDr (%)	5	6	7	6	7	6
	SDR (µg/kg)	12.6	6.72	5.45	2.49	3.97	1.92
	RSDR (%)	9	9	13	8	10	10
	Rec (%)	104	110	108	88	100	90
	HorRat Values	132	68	38.9	36	38.2	20.8
**FB1**	Assigned Value (µg/kg)	275	4262	n.d	72	121	31.1
	No. of Laboratories	20	18	-	20	20	20
	No. of Outliers	0	0	-	1	0	0
	No. of Accepted Results	20	18	-	19	20	20
	Mean (µg/kg)	291	4735	-	76.5	125	33.1
	SDr (µg/kg)	14.9	258	-	4.32	8.15	3.14
	RSDr (%)	5	5	-	6	7	9
	SDR (µg/kg)	35.2	541	-	11.9	18.6	5.53
	RSDR (%)	12	11	-	16	15	17
	Rec (%)	106	111	-	106	103	106
	HorRat Values	0.6	0.9	-	0.7	0.7	0.8
**FB2**	Assigned Value (µg/kg)	223	1299	n.d	72	130	44.2
	No. of Laboratories	20	19	-	20	20	20
	No. of Outlier	0	0	-	2	2	2
	No. of Accepted Results	20	19	-	18	18	18
	Mean (µg/kg)	245	1500	-	74.8	134	41.1
	SDr (µg/kg)	10	72.2	-	5.46	7.45	4.17
	RSDr (%)	4	5	-	7	6	10
	SDR (µg/kg)	25	180	-	7.25	10	4.63
	RSDR (%)	10	12	-	10	7	11
	Rec (%)	110	115	-	104	103	93
	HorRat Values	0.5	0.8	-	0.4	0.3	0.5
**Total FBs**	Assigned Value (µg/kg)	485	5528	n.d.	150	245	75.3
	No. of Laboratories	20	18	-	20	20	20
	No. of Outliers	0	0	-	2	1	0
	No. of Accepted Results	20	18	-	18	19	20
	Mean (µg/kg)	536	6233	-	152	263	76
	SDr (µg/kg)	18.6	291	-	8.9	12.3	5.6
	RSDr (%)	3	5	-	6	5	7
	SDR (µg/kg)	51.5	588	-	16.1	26.7	11.3
	RSDR (%)	10	9	-	11	10	15
	Rec (%)	110	113	-	101	107	101
	HorRat Values	0.5	0.8	-	0.5	0.5	0.7

**Table 4:** Method performance evaluation for AFM1 in two milk powder samples (S13 and S14).

		S13	S14
**AFM1**	**Assigned Value (µg/kg)**	0.1121	0.0342
	**No. of Laboratories**	20	16
	**No. of Outliers**	2	2
	**No. of Accepted Results**	18	14
	**Mean (µg/kg)**	0.127	0.0333
	**SDr (µg/kg)**	0.013	0.0073
	**RSDr (%)**	10	22
	**SDR (µg/kg)**	0.017	0.0087
	**RSDR (%)**	13	26
	**Rec (%)**	113	97
	**HorRat Values**	0.6	1.2

**Table 5:** Fitness-for-purpose of the method with regards to EU regulations for foods intended for infants and young children.

Mycotoxins	ML (µg/kg) ^a^	Lowest Validated Level (µg/kg)	RSDr (%)	RSDR (%)	Rec%
**AFB1**	0.1	0.084	12	18	99
**OTA**	0.5	0.44	8	13	99
**DON**	200	43.6	8	9	97
**ZEN**	20	8.8	8	13	92
**T-2 + HT-2**	15 ^b^	18.6	6	10	90
**FBTOT**	200	76.2	7	15	101
**AFM1**	0.025 ^c^	0.0117	10	13	113

^a^ Commission Regulation (EC) No. 1881/2006; ^b^ Commission Regulation (EC) No. 165/2013; ^c^ refers to the products ready to use (marketed as such or after reconstitution as instructed by the manufacturer).

## Supplementary Materials

The following are available online at https://www.mdpi.com/2072-6651/11/11/658/s1: Document 1: Determination of mycotoxins by liquid chromatography-tandem mass spectrometry (LC-MS/MS), standard operation procedure for the method validation study, 3 July 2019.
