# Supplementary material for: Multiple Mycotoxins Determination in Food by LC-MS/MS: An International Collaborative Study
Source: Toxins (Basel). 2019 Nov 12;11(11):658. doi: 10.3390/toxins11110658 (PMC6891721; doi:10.3390/toxins11110658)
Supplement: Supplementary file 1 [file toxins-11-00658-s001.pdf]

Determination of mycotoxins by liquid chromatography-tandem mass spectrometry (LC-MS/MS)

Standard operation procedure for the method validation study

03.06.2019

Contents

Page

Introduction 3

1 Scope 4

2 Principle 4

3 Reagents 5

4 Apparatus 12

5 Sampling 13

6 Procedure 14

7 LC-MS/MS analysis 16

8 Calculations 18

Annex A : Overview of the sample preparation 20

Annex B : (informative) Example: Instrumental LC-MS/MS conditions 21

B.2 MS conditions 22

Bibliography 24

Introduction

Mycotoxins are toxic fungal metabolites occurring in a wide range of foodstuffs such as cereals, nuts, spices, fruits, oil seeds, or coffee. Among several hundreds of mycotoxins identified so far, a few are of concern from a food safety perspective.

Trichothecenes are a group of mycotoxins produced particularly by *Fusarium* moulds. They are often grouped as groups A and B which are characterized by specific structural features. **T-2 toxin (T-2)** and **HT-2 toxin (HT-2)** are common type A representatives, while **Deoxynivalenol (DON)** is common type B representative. Trichothecenes initiate a wide range of effects in animal and human, such as reduced consumption of feed, skin irritation, diarrhea, multiple haemorrhages and immunosuppressive effects.

Ochratoxin A (OTA) is produced by several *Aspergillus* and *Penicillium* species in semitropical and temperate climates. OTA is a potent nephrotoxin and hepatoxin with teratogenic, mutagenic, carcinogenic and immunosuppressive effects even at trace levels.

Zearalenone (ZEN) is a nonsteroidal estrogenic mycotoxin, which is frequently produced by *Fusarium* species. This mycotoxin exhibits striking estrogenic and anabolic properties in human and animal resulting in severe effects on the reproductive system.

Fumonisins (FBs) are a group of structurally related toxic metabolites produced by *Fusarium* species. They occur at high incidence in corn and corn products all over the world. **Fumonisin B1 (FB1)** is the predominant metabolite, followed by **Fumonisin B2 (FB2)** and Fumonisin B3. **FB1** is known to cause a range of species-specific toxic responses, including leukoencephalomalacia in horses, pulmonary oedema in swine, and hepatosis and nephrotoxicity in rodents. **FB1** is carcinogenic for female mice and male rats.

Aflatoxins (AFLAs) are naturally related mycotoxins produced by numerous *Aspergillus* species. About 20 compounds have been described, but only Aflatoxin B1 **(AFLA B1)**, Aflatoxin B2 **(AFLA B2)**, Aflatoxin G1 **(AFLA G1)** and Aflatoxin G2 **(AFLA G2)** occurs in food commodities. **AFLA B1** is the most toxic aflatoxin. When lactating cattle and other animals ingest aflatoxins in contaminated feed, toxic metabolites such as Aflatoxin M1 **(AFLA M1)** can be formed and transmitted to milk. This hydroxylated form is a potentially important contaminant in dairy products. The International Agency for Research on Cancer (IARC) has classified aflatoxins as carcinogenic to humans (Group 1).

To protect consumer health, maximum levels (MLs) for mycotoxins in foodstuffs have been established worldwide. Particularly, the European legislation has established MLs for AFLAs, OTA, ZEN, FBs and DON and recently indicative levels for T-2 and HT-2 toxins [1,2].

WARNING 1 — Suitable precaution and protection measures need to be taken when carrying out working steps with harmful chemicals. The European Union hazardous substances ordinance (EU) 1907/2006 [3], should be taken into account as well as appropriate National statements.

WARNING 2 — The use of this document can involve hazardous materials, operations and equipment. This document does not purport to address all the safety problems associated with its use. It is the responsibility of the user of this document to establish appropriate safety and health practices and determine the applicability of regulatory limitations prior to use.

1 Scope

This document describes a multiresidue method for the quantitative determination of 12 mycotoxins in foods (cereals, infant food, nuts, dried fruits, spices, milk powder…) by liquid chromatography-tandem mass spectrometry (LC-MS/MS). The mycotoxins considered are: ochratoxin A (OTA); aflatoxins B1, B2, G1, G2 and M1 (AFLAs); zearalenone (ZEN); fumonisins B1 and B2 (FBs); T-2 and HT-2 toxins and deoxynivalenol (DON).

The range of concentrations covered for each mycotoxin is described hereafter and ensures quantification at or below EU regulatory limits [1,2]:

- Ochratoxin A: 0.125 – 32 µg/kg
- Aflatoxins B1, B2, G1, G2, M1: 0.025 – 32 µg/kg
- Zearalenone: 5 – 640 µg/kg
- Fumonisins B1 and B2: 12.5 – 1600 µg/kg
- T-2 and HT-2 toxins: 6.25 – 800 µg/kg
- Deoxynivalenol: 12.5 – 1600 µg/kg

2 Principle

Mycotoxins are extracted according to the European Norm EN 15662:2008 (QuEChERS protocol) [4] with some modifications. The protocol involves an initial single phase extraction with water and acidified acetonitrile, followed by liquid-liquid partition by addition of magnesium sulphate and sodium chloride. The resulting acetonitrile supernatant obtained is then defatted with hexane. Depending on the mycotoxin/matrix combination and the sensitivity required for Aflatoxins and OTA, the sample extracts can then be submitted to two different clean-up procedures, named "QuEChERS" and "IAC":

- "QuEChERS": Generic clean-up for all mycotoxins potentially present in cereals when an improved sensitivity for AFLAs and OTA is not required. An aliquot of the acetonitrile supernatant is evaporated to dryness and reconstituted in methanol-water prior injection onto the LC-MS/MS system.
- "IAC": Specific clean-up for AFLAs and OTA for sensitivity purpose in infant foods (e.g. infant cereals, infant formula) and "difficult" matrices (e.g. spices, dried fruits and nuts). An aliquot of the acetonitrile supernatant is first diluted in a phosphate buffered saline (PBS) solution and the whole extract is then applied onto an immunoaffinity column (IAC) containing antibodies specific to AFLAs and OTA. The IAC is washed with water and the toxins are eluted with methanol. The eluate is evaporated to dryness and reconstituted in methanol-water prior injection onto the LC-MS/MS system.

Positive identification of mycotoxins in the sample must fulfill the confirmation criteria defined in the SANTE/12089/2016 document for mass spectrometry analysis [5]. Quantification is performed by the isotopic dilution approach using ^{13}C-labeled mycotoxins as internal standards (ISs).

3 Reagents

Use only reagents of recognized analytical grade and water complying with grade 1 of EN ISO 3696, unless otherwise specified. Solvents shall be of quality for LC-MS analysis, unless otherwise specified. Commercially available solutions with equivalent properties to those listed may be used.

3.1 Chemicals

3.1.1 **Water,** HPLC grade

3.1.2 **Water,** LC-MS grade or water of grade 1 as defined in EN ISO 3696

3.1.3 **Methanol,** LC-MS grade

3.1.4 **Acetonitrile,** HPLC grade

3.1.5 **Formic Acid,** analytical reagent grade

3.1.6 **Ammonium formate,** LC-MS grade

3.1.7 **Acetic acid glacial,** analytical reagent grade

3.1.8 ***n*-hexane,** HPLC grade

3.1.9 **Magnesium sulphate ($MgSO_4$) anhydrous,** analytical reagent grade

3.1.10 **Sodium chloride (NaCl),** analytical reagent grade

3.1.11 **Potassium chloride (KCl),** analytical reagent grade

3.1.12 **Potassium dihydrogen phosphate (KH_2PO_4),** analytical reagent grade

3.1.13 **Disodium hydrogen orthophosphate (Na_2HPO_4),** analytical reagent grade

3.1.14 **Sodium hydroxide (NaOH),** analytical reagent grade.

3.1.15 **Hydrochloric acid (HCl) solution,** 37 %, analytical reagent grade

3.2 Mycotoxins Unlabelled Standard Stock Solutions

3.2.1 **Aflatoxin B1 (AFLA B1),** *e.g.* crystalline, as a film or as certified standard solution

3.2.2 **Aflatoxin B2 (AFLA B2),** *e.g.* crystalline, as a film or as certified standard solution

3.2.3 **Aflatoxin G1 (AFLA G1),** *e.g.* crystalline, as a film or as certified standard solution

3.2.4 **Aflatoxin G2 (AFLA G2),** *e.g.* crystalline, as a film or as certified standard solution

3.2.5 **Aflatoxin M1 (AFLA M1),** *e.g.* crystalline, as a film or as certified standard solution

3.2.6 **Deoxynivalenol (DON),** *e.g.* crystalline, as a film or as certified standard solution

3.2.7 **T-2 Toxin (T-2),** *e.g.* crystalline, as a film or as certified standard solution

3.2.8 **HT-2 Toxin (HT-2),** *e.g.* crystalline, as a film or as certified standard solution

3.2.9 **Zearalenone (ZON),** *e.g.* crystalline, as a film or as certified standard solution

3.2.10 **Fumonisin B1 (FB1),** *e.g.* crystalline, as a film or as certified standard solution

3.2.11 **Fumonisin B2 (FB2),** *e.g.* crystalline, as a film or as certified standard solution

3.2.12 **Ochratoxin A (OTA),** *e.g.* crystalline, as a film or as certified standard solution

3.3 Mycotoxins Labelled Standard Stock Solutions

3.3.1 **($^{13}C_{17}$)-Aflatoxin B1,** *e.g.* 0.5 µg/mL in acetonitrile

3.3.2 **($^{13}C_{17}$)-Aflatoxin B2,** *e.g.* 0.5 µg/mL in acetonitrile

3.3.3 **($^{13}C_{17}$)-Aflatoxin G1,** *e.g.* 0.5 µg/mL in acetonitrile

3.3.4 **($^{13}C_{17}$)-Aflatoxin G2,** *e.g.* 0.5 µg/mL in acetonitrile

3.3.5 **($^{13}C_{17}$)-Aflatoxin M1,** *e.g.* 0.5 µg/mL in acetonitrile

3.3.6 **($^{13}C_{15}$)-Deoxynivalenol,** *e.g.* 25 µg/mL in acetonitrile

3.3.7 **($^{13}C_{24}$)-T-2 Toxin,** *e.g.* 25 µg/mL in acetonitrile

3.3.8 **($^{13}C_{22}$)-HT-2 Toxin**, *e.g.* 25 µg/mL in acetonitrile

3.3.9 **($^{13}C_{18}$)-Zearalenone,** *e.g.* 25 µg/mL in acetonitrile

3.3.10 **($^{13}C_{34}$)-Fumonisin B1,** *e.g.* 25 µg/mL in acetonitrile/water (50+50)

3.3.11 **($^{13}C_{34}$)-Fumonisin B2,** *e.g.* 10 µg/mL in acetonitrile/water (50+50)

3.3.12 **($^{13}C_{20}$)-Ochratoxin A,** *e.g.* 10 µg/mL in acetonitrile

3.4 Unlabelled Working Standard Solutions – NOT NEEDED FOR THE COLLABORATIVE STUDY

The individual solutions are either prepared by dissolving neat (solid) standards in an appropriate solvent, or from individual stock solutions purchased as such. The mycotoxins covered in this standard dissolve well in acetonitrile, with the exception of fumonisins for which acetonitrile/water (50+50, v+v) is recommended for preparing individual stock solutions.

Prepare the unlabeled working standard solutions as described hereafter by combining the appropriate volumes of individual mycotoxin solutions, using the appropriate pipets (4.1) and the mentioned solvent.

These solutions are used to build the calibration curve (3.6).

3.4.1 Aflatoxins composite working standard solution (AFLA B1, B2, G1 and G2), 0.1 µg/mL in acetonitrile

3.4.2 Aflatoxins composite working standard solution (AFLA B1, B2, G1 and G2), 0.01 µg/mL in acetonitrile

3.4.3 AFLA M1 working standard solution, 0.1 µg/mL in acetonitrile

3.4.4 AFLA M1 working standard solution, 0.01 µg/mL in acetonitrile

3.4.5 [DON, T-2, HT-2, ZEN]-composite working standard solution, in acetonitrile at concentrations given in **Table 1**.

Table 1. **[DON, T-2, HT-2, ZEN]-composite working standard solution concentrations**

Compound	Concentration [µg/mL]
DON	5
T-2	2.5
HT-2	2.5
ZEN	2

3.4.6 Fumonisins composite working standard solution (FB1 + FB2), 5 µg/mL in acetonitrile-water (50+50, v+v)

3.4.7 OTA working standard solution, 0.1 µg/mL in methanol-water (85+15, v+v)

3.5 Isotopically Labelled Working Standard Solutions - PROVIDED AS READY-TO-USE FOR THE COLLABORATIVE STUDY

Isotopically labelled mycotoxins are commercially available as certified standard solutions. Prepare the labelled working standard solutions as described hereafter by combining the appropriate volumes of individual labelled mycotoxin solutions, using the appropriate pipets (4.1) and the mentioned solvent.

These solutions are used to build the calibration curve (3.6) and to spike each test portions at the beginning of the sample preparation (6.2) for quantification purpose.

3.5.1 ^{13}C-Aflatoxins composite working standard solution (($^{13}C_{17}$)-AFLA B1, ($^{13}C_{17}$)-AFLA B2, ($^{13}C_{17}$)-AFLA G1 and ($^{13}C_{17}$)-AFLA G2), 0.1 µg/mL in acetonitrile

3.5.2 ($^{13}C_{17}$)-AFLA M1 working standard solution, 0.1 µg/mL in acetonitrile

3.5.3 ^{13}C-[DON, T-2, HT-2, ZEN]-composite working standard solution, in acetonitrile at concentrations given in Table 2.

Table 2. ^{13}C-[DON, T-2, HT-2, ZEN]-composite working standard solution concentrations

Compound	Concentration [µg/mL]
($^{13}C_{15}$)-DON	5
($^{13}C_{24}$)-T-2	2.5
($^{13}C_{22}$)-HT-2	2.5
($^{13}C_{18}$)-ZEN	2

3.5.4 ^{13}C-Fumonisins composite working standard solution (($^{13}C_{34}$)-FB1, ($^{13}C_{34}$)-FB2), 10 µg/mL in acetonitrile-water (50+50, v+v)

3.5.5 ($^{13}C_{20}$)-OTA working standard solution, 0.1 µg/mL in methanol-water (85+15, v+v)

3.6 Standard Solutions for External Calibration Curve – PROVIDED AS READY-TO-USE FOR THE COLLABORATIVE STUDY

Note: The same batch of IS must be used for both making the calibration solutions and spiking the extracts as described in the extraction procedure (6.2). Any deviation from this may lead to wrong quantification.

Into nine separate 15-mL polypropylene tubes, prepare the standard solutions for calibration as described in **Table 3**. Concentration of each mycotoxin in each calibrant solution is given in **Table 4**. Transfer these solutions into glass vials and store them at -20 °C away from light for up to 3 months.

Table 3. Pipetting scheme for the preparation of the calibration solutions

Volume in µL	CAL 0	CAL 1	CAL 2	CAL 3	CAL 4	CAL 5	CAL 6	CAL* 7	CAL* 8
Aflatoxin mix (3.4.1)	0	-	-	-	20	40	160	320	640
Aflatoxin mix (3.4.2)	-	10	20	40	-	-	-	-	-
AFLA M1 (3.4.3)	0	-	-	-	20	40	160	320	640
AFLA M1 (3.4.4)	-	10	20	40	-	-	-	-	-
[DON, T-2, HT-2, ZEN]-mix (3.4.5)	0	10	10	20	40	80	160	320	640
Fumonisins mix (3.4.6)	0	10	10	20	40	80	160	320	640
^{13}C-Aflatoxins mix (3.5.1)	20	40	20	20	20	20	20	20	20
^{13}C-AFLA M1(3.5.2)	20	40	20	20	20	20	20	20	20
^{13}C-[DON, T-2, HT-2, ZEN]-mix (3.5.3)	20	40	20	20	20	20	20	20	20
^{13}C-Fumonisins mix (3.5.4)	20	40	20	20	20	20	20	20	20
Evaporate to dryness under a stream of nitrogen at 40°C then add the following standards:									
OTA (3.4.7)	0	5	5	10	20	40	160	320	640
^{13}C-OTA (3.5.53.5.1)	20	40	20	20	20	20	20	20	20
MeOH-H_2O (15+85) (3.7.2)	1980	3955	1975	1970	1960	1940	1820	1660	1340
Sonicate the calibrants CAL 0 to CAL 8 for about 1 minute									

Note: OTA solutions are added after the evaporation step to avoid unpredictable losses of OTA and ^{13}C-OTA during the evaporation step.

Note*: The calibration range can be extended for quantification of highly contaminated samples. Typically, CAL 7 and CAL 8 can be prepared as described in **Table 3** to extend the range by a factor of 2 and a factor of 4, respectively.

Table 4. Calibration solutions concentrations (expressed in ng/mL)

In ng/mL	CAL 0	CAL 1	CAL 2	CAL 3	CAL 4	CAL 5	CAL 6	CAL 7	CAL 8
AFLAs	0	0.025	0.1	0.2	1	2	8	16	32
DON	0	12.5	25	50	100	200	400	800	1600
T-2 & HT-2	0	6.25	12.5	25	50	100	200	400	800
ZEN	0	5	10	20	40	80	160	320	640
FBs	0	12.5	25	50	100	200	400	800	1600
OTA	0	0.125	0.25	0.5	1	2	8	16	32
^{13}C-AFLAs	1	1	1	1	1	1	1	1	1
^{13}C-DON	50	50	50	50	50	50	50	50	50

^{13}C-T-2 &^{13}C-HT-2	25	25	25	25	25	25	25	25	25
^{13}C-FBs	100	100	100	100	100	100	100	100	100
^{13}C-OTA	1	1	1	1	1	1	1	1	1

3.7 Solutions and Salt Mixtures for Sample Preparation

3.7.1 Extraction solvent: acetic acid 5 mL/L in acetonitrile

Into a 1-L volumetric flask, mix 500 mL of acetonitrile (3.1.4) and 5 mL of acetic acid (3.1.7). Complete to volume with acetonitrile and mix well. Store this solution at room temperature for no longer than 3 months.

3.7.2 Methanol-water (15+85, v+v)

Into a 100-mL volumetric flask, mix 15 mL of methanol (3.1.3) with 85 mL of water (3.1.1). Store this solution at room temperature for no longer than 3 months.

3.7.3 Phosphate buffered saline (PBS) solution preparation - PROVIDED AS READY-TO-USE FOR THE COLLABORATIVE STUDY

3.7.3.1 Sodium hydroxide solution, 0,1 mol/L

Into a 100-mL volumetric flask, add 0,4 g sodium hydroxide (3.1.14). Dissolve with water and fill up to the mark with water.

3.7.3.2 Hydrochloric acid solution, 0,1 mol/L

Into a 1-L volumetric flask, add 8,2 ml of hydrochloric acid solution (3.1.15). Complete to volume with water.

3.7.3.3 PBS solution, pH 7.3 ± 0.2

Weigh 0,20 g of potassium chloride (3.1.11), 0,20 g of potassium dihydrogen phosphate (3.1.12), 1,15 g of disodium hydrogen orthophosphate (3.1.13) and 8,00 g of sodium chloride (3.1.10) to the nearest 0,01 g and transfer into a 1 L volumetric flask. Dissolve in water and add 900 ml of water.
After dissolution adjust the pH to 7,3 with either hydrochloric acid solution (3.7.3.2) or sodium hydroxide solution (3.7.3.1), then fill up to the mark with water.

Alternatively, a PBS solution with equivalent properties may be prepared from commercially available PBS material.

3.7.4 QuEChERS partition salts - PROVIDED AS READY-TO-USE FOR THE COLLABORATIVE STUDY

Into a 15-ml polypropylene tube, weigh 4,0 g ± 0,2 g $MgSO_4$ (3.1.9) and 1,00 g ± 0,05 g NaCl (3.1.10).

Alternatively, a ready-to-use $MgSO_4$ – NaCl (4+1) salt mixture is an example of a suitable product commercially available.

3.8 Solutions for LC-MS/MS

3.8.1 Mobile phase (A): formic acid (1.5 mL/L) in water containing 10 mM of ammonium formate

Into a 1-L volumetric flask, dissolve 0.630 g ± 0.005 g of ammonium formate (3.1.6) with about 500 mL of water (3.1.2). Add 1.5 mL of concentrated formic acid (3.1.5) and complete to the mark with water (3.1.2). Mix well and transfer into a LC bottle.

Store this solution at room temperature for no longer than 1 week.

3.8.2 Mobile phase (B): formic acid (0.5 mL/L) in methanol

Into a 500-mL volumetric flask, add 250 mL of methanol (3.1.3) and 250 µL of concentrated formic acid (3.1.5). Complete to the mark with methanol (3.1.3). Mix well and transfer into a LC bottle.

Store this solution at room temperature for no longer than 1 week.

3.8.3 Injection flush port: Water–Methanol (1+1, v+v)

Into a 1-L volumetric flask, add 500 ml of methanol (3.1.3). Dilute to volume with water (3.1.2). Transfer into a LC bottle.

Store at room temperature for no longer than 3 months.

4 Apparatus

Standard laboratory apparatus may be used (graduated cylinders, glass funnels, beakers, pipette, etc.) and, in particular, the following.

4.1 **Calibrated volumetric pipettes,** suitable for organic solvent in the range 5 µl to 1 ml

4.2 **50-mL conical polypropylene centrifuge tube,** with caps

4.3 **15-mL conical polypropylene centrifuge tube,** with caps

4.4 **1.5-mL polypropylene microcentrifuge tube**

4.5 **HPLC glass vial,** 1.5 mL with cap

4.6 **Adjustable mechanical vertical** or **horizontal shaker,** capable to shake at 300 rpm

4.7 **Vortex mixer**

4.8 **Ultrasonic water bath**

4.9 **Laboratory blender,** e.g. Ultra Turrax®[1].

4.10 **Laboratory balance,** with a mass resolution of 0.01 g

4.11 **Analytical balance,** with a mass resolution of 0.0001 g

4.12 **Centrifuge,** with rotors adapted for 15-mL and 50-mL polypropylene tubes, with at least 4'000 x *g* speed

4.13 **Centrifuge,** with rotors adapted for 1.5-mL polypropylene tubes, with at least 8'400 x *g* speed

4.14 **Sample concentrator,** with temperature control and gas supply

4.15 **Vacuum manifold for SPE clean-up,** with taps

4.16 **Polypropylene reservoirs (approx. 25 mL),** adapted for SPE columns

4.17 **Disposable syringe,** 5 mL

4.18 **Plastic Pasteur pipette,** non-sterile, 7 mL

4.19 **Immunoaffinity columns (IAC)** [2]

The affinity column contains antibodies raised against aflatoxins B1, B2, G1, G2 and OTA. A cross-reactivity with Aflatoxin M1 is also suitable to analyze Aflatoxin M1 in milk based product samples.

---

[1] Ultra Turrax® is a trade name of a product commercially available from various suppliers. This information is given for the convenience of users of this European Standard and does not constitute an endorsement by CEN of the product named. Equivalent products may be used if they can be shown to lead to the same results.

[2] AFLAOCHRA PREP® column from R-biopharm, is an example of a suitable product available commercially. This information is given for the convenience of users of this European Standard and does not constitute an endorsement by CEN of the product named. Equivalent products may be used if they can be shown to lead to the same results.

4.20 LC-MS/MS apparatus, comprising the following

4.20.1 **LC pump,** capable of delivering a binary gradient and operating at speed of 0.5 ml/min.

4.20.2 **Degasser,** optional, for degassing HPLC mobile phases.

4.20.3 **Autosampler,** capable of being temperature controlled at 10 °C and injecting volumes of 5 µl with sufficient accuracy.

4.20.4 **Column oven**, capable to operate at 50 °C ± 1 °C.

4.20.5 **Triple stage mass spectrometer** (*e.g* triple quadrupole or quadrupole linear ion trap) equipped with an electrospray ionization (ESI) interface operating both on positive and negative mode.

4.20.6 **U(H)PLC reversed phase column**[3], capable to retain the first eluting analyte at at least twice the retention time corresponding to the void volume of the column.

4.20.7 **HPLC Pre-column,** optional, with the same stationary phase material as the HPLC column.

4.20.8 **Computer based control and data processing system**

5 Sampling

Sampling is not part of the method specified in this document. The Commission Regulation (EC) No 401/2006 [6] can be used as a reference.

It is important that the laboratory receives a sample which is representative and has not been damaged or changed during transport or storage. Powdered samples must be homogenized before taking test portions. This could be achieved by transferring the whole sample into a container of capacity about twice that of the laboratory sample volume, and thoroughly mixing by repeatedly shaking and inverting the container. Alternatively, the powdered laboratory sample could be directly homogenised into its original container by means of a spoon before taking a test portion.

[3] Waters BEH C18 (2.1 x 100 mm, 1.7 µm) is an example of a suitable product available commercially. This information is given for the convenience of users of this European Standard and does not constitute an endorsement by CEN of the product named. Equivalent products may be used if they can be shown to lead to the same results.

6 Procedure

6.1 Test portions preparation

6.1.1 Cereals, infant cereals

Into a 50-mL polypropylene tube, weigh a test portion of 5.00 g of the laboratory sample to the nearest 0.05 g.

6.1.2 Milk powders, nuts, spices, dried fruits

Into a 50-mL polypropylene tube, weigh a test portion of 2.00 g of the laboratory sample to the nearest 0.02 g.

6.2 Spiking of ^{13}C-labelled mycotoxins used as internal standards

Note: Robustness of the method is not affected as long as the same mycotoxin standard solutions are used for both preparing calibration standards and spiking test portions.

Note: The choice of the IS depends on the final purpose of the analysis.

Spike each test portion with 50 µL of individual and/or composite working standard solutions of ^{13}C-labeled mycotoxins used as internal standards (IS) as shown in **Table 5**.

Table 5. Spiking experiments of IS solutions

IS	Spiking Level, µL	Amount added on test portion, ng
^{13}C-Aflatoxins mix, 0.1 µg/mL (3.5.1)	50	5
^{13}C-AFLA M1, 0.1 µg/mL (3.5.2)	50	5
^{13}C-[DON, T-2, HT-2, ZEN]-mix (3.5.3) *	50	250, 125, 125, 100
^{13}C-Fumonisin mix, 10 µg/mL (3.5.4)	50	500
^{13}C-OTA, 0.1 µg/mL (3.5.5),	50	5

** Concentration for ($^{13}C_{15}$)-DON, ($^{13}C_{24}$)-T-2, ($^{13}C_{22}$)- HT-2 and ($^{13}C_{18}$)-ZEN are 5, 2.5, 2.5 and 2 µg/mL, respectively*

6.3 Extraction

Add 10 mL of water (3.1.1) and shake vigorously by hand until the whole sample is completely dispersed in solution.

Add 10 mL of acidified acetonitrile (3.7.1), close the tubes and shake vigorously by hand for at least 5 s. Shake on a mechanical shaker for approximately 10 min at approximately 300 rpm.

Open the tubes and add 5.0 g ± 0.2 g of the magnesium sulphate-sodium chloride salt mixture (3.7.4) to the slurry. Close the tubes and immediately shake for a few seconds to avoid formation of lumps of magnesium sulphate. Shake the tube vigorously for about 1 min by hand or on a mechanical shaker. Centrifuge the tubes at 4000 x *g* at room temperature for approximately 10 min.

6.4 General clean-up procedure: removal of co-extracted fat

Into a 15-mL polypropylene tube, transfer 5 mL of the supernatant acetonitrile phase (6.3) and add 5 mL of *n*-hexane (3.1.8). Shake for approximately 10 min on a mechanical shaker.

Centrifuge at 4000 x *g* for approximately 1 min to allow an efficient phase separation

Discard the *n*-hexane (upper phase) by using a plastic Pasteur pipette (4.18).

6.5 Specific clean-up procedures ("QuEChERS" or "Immunoaffinity column (IAC)")

At this point of the method, the sample extract can be divided in two portions and submitted to two different clean-up protocols according to the general scheme presented in **Annex A**:

- 1st option: all analytes are directed to the "QuEChERS" procedure (cereals-based samples).
- 2nd option: for AFLAs and OTA only, when extra sensitivity is needed (food intended for infants and young children) and for difficult matrices (e.g. spices, dried fruits and nuts), the sample extract is directed to the "IAC" procedure.

6.5.1 "QuEChERS" (generic clean-up for all mycotoxins)

Transfer a 1-mL aliquot of the defatted acetonitrile phase (lower phase, 6.4) into a new 15-mL polypropylene tube.

Evaporate the extract to dryness under a stream of nitrogen at about 40 °C.

Reconstitute the residue with 75 µL of methanol (3.1.3) and sonicate for about 1 min to re-suspend the residue. Add 425 µL of water (3.1.1) and mix for about 5 s using a vortex mixer. Transfer the resulting mixture in a 1.5-mL polypropylene microcentrifuge tube and centrifuge for about 10 min at 8′500 x *g* at room temperature.

Transfer the supernatant into a LC amber glass vial and proceed with LC-MS/MS analysis.

6.5.2 "IAC" (specific clean-up for OTA and AFLAs)

Transfer a 2-mL aliquot of the defatted acetonitrile phase (lower phase, 6.4) into a new 50-mL polypropylene tube. Dilute approximately to the 25-mL mark with the PBS solution (3.7.3.3) and mix well.

Allow the IAC to reach room temperature prior to use. Connect the IAC (4.19) to the vacuum manifold (4.15) and attach a SPE tube adapter with a reservoir with a minimum capacity of 25 mL at the top of the IAC.

Loading step:

Transfer the whole diluted extract (25 mL) into the IAC reservoir and pass it through the IAC by applying a gentle vacuum to get a flow rate of approximately 1-2 drops/s.

Washing Step:

Wash the column with 20 mL of water (3.1.1) at an approximate flow rate of 1-2 drops/s. Remove the reservoir and dry the column.

Elution Step:

Place a 15-mL polypropylene tube beneath the column and apply at first 800 µL of methanol (3.1.3). Slowly elute the first drop and stop the elution. Let the methanol diffuse for approximately 3 min. Backflush the methanol with a 5-mL disposable syringe attached to the top of the column while pushing it through into the 15-mL polypropylene tube. Repeat the back flushing at least twice (the backflushing step should be carefully conducted to ensure complete denaturation of antibodies). Let

elute the remaining methanol by gravity. Pass two more 800-µL portions of methanol and let elute by gravity. Cap the 15-mL polypropylene tube and vortex for about 10 s.

Final Treatment:

Evaporate the extract to dryness under a stream of nitrogen at about 40 °C. Reconstitute the residue with 30 µL of methanol (3.1.3) and sonicate for about 1 min to re-suspend the residue. Add 170 µL of water (3.1.1) and mix for about 5 s using a vortex mixer. Transfer the resulting mixture in a 1.5-mL polypropylene microcentrifuge tube and centrifuge for about 10 min at 8'500 x *g* at room temperature.

Transfer the supernatant into a HPLC glass vial and proceed with LC-MS/MS analysis.

7 LC-MS/MS analysis

7.1 LC conditions

Using the column (4.20.6) and the mobile phases A and B (3.8.1 and 3.8.2) specified in this document, the following LC conditions are adapted for mycotoxin analysis:

- Injection volume: 10 µL
- Column Temperature: 50 °C
- Flow rate: 400 µL/min
- Gradient: as shown in **Table 6**

Table 6. Example of LC program for multi-mycotoxin analysis

Time [min]	A [%]	B [%]
0	85	15
0.3	85	15
4	0	100
7	0	100
7.1	85	15
10	85	15

7.2 MS Conditions

The values given in Annex B.1.2 need to be checked and optimized for each instrument. It is recommended to optimize MS parameters by syringe-infusing each individual mycotoxin standard solution in electrospray ionisation (ESI) mode. At least two transition reactions per compound must be monitored for each mycotoxin and its relative internal standard.
The most intense transition reaction may differ depending on the instrument and eluents used in LC (*e.g.* protonated molecules or sodium/ammonium adducts in ESI+, deprotonated molecules or acetate/formate adducts in ESI-).

7.3 Injection sequence

Start a batch of analysis by injecting a methanol-water (15+85) (3.7.2) solution to stabilize the LC-MS/MS system and to check for any contamination of the system.

Inject the calibration solutions from CAL 0 to CAL 6 (3.6) and carefully check that all toxins and their respective ISs are visible at the lowest calibration level (CAL 1). Inject a methanol-water (15+85) (3.7.2) solution to ensure that there is no carry over.

Inject the sample extracts (6.5.1 and/or 6.5.2). Inject regularly the methanol-water (15+85) (3.7.2) solution to check for any carry over.

End the sequence by re-injecting at least one calibration solution (3.6) to ensure system stability. CAL 7 and CAL 8 can be injected at the end of the sequence. Rinse the column afterwards by injecting the methanol-water solution (3.7.2) at least twice.

7.4 Data Treatment

Process the data using the appropriate integration software. Peaks areas are used for subsequent calculations. Check peak area assignment and integration for the measured transition reaction and adjust if necessary.

8 Calculations

8.1 Identification and confirmation

Mycotoxins are considered as positively identified in the sample when all the confirmation criteria defined in the SANTE/12089/2016 document [5] are fulfilled:

- A signal is visible at least at two diagnostic transition reactions selected for each mycotoxin and each corresponding IS.
- The retention time of the analyte in the sample extract should correspond to that of the average of the calibration standards measured in the same sequence with a tolerance of ±0.2 min.
- The retention time of the analyte should correspond to that of its labelled internal standard with a tolerance of ±0.05 min.
- The peak area ratio from the different transition reactions recorded for each analyte is ± 30%.

8.2 Calibration curve

Quantification is performed by the isotopic dilution approach using ^{13}C-labeled mycotoxins as internal standard (IS).

Draw the calibration curve (Area Ratio = [(Concentration Ratio) x Slope] + Intercept) by plotting peak area ratio of each mycotoxin and its IS (= y axis) against concentration ratio of each mycotoxin and its IS (= x axis) using calibration solutions from CAL 0 to CAL 6.

Calculate the slope and the intercept by linear regression and check that the calibration curve is linear:

- Regression coefficient R^2 should be higher than 0.99.
- The deviation of the back-calculated concentration of the calibrants standards from the true concentration, using the calibration curve, should not be more than ±20%.

Note: In order to improve the precision on the low calibration points, it is advisable to use a 1/x or $1/x^2$ weighing factor for the calibration curve. Alternatively, ensure that the confidence interval around the intercept contains 0 and force the regression line through the origin (*i.e.* Intercept = 0).

Note: CAL 7 and CAL 8 (3.6) are only to be considered in case of highly contaminated sample whene mycotoxin levels are out of the calibration range.

8.3 Calculations

Calculate the mass fraction of each analyte, w_a, in microgram per kilogram (µg/kg) in the sample using the following equation:

$$w_a = \frac{\left(\frac{A_a}{A_{is}}\right) - I}{S} \times \frac{m_{is}}{m_a}$$

Where:

w_a = mass fraction of analyte in sample, in µg/kg

A_a = peak area of a given analyte

A_{is} = peak area of the corresponding IS

I = intercept of the regression line for the transition reaction used for quantitation
S = slope of the regression line for the transition reaction used for quantitation
m_a = mass of the test portion, in g (either 2.0 g or 5.0 g)
m_{is} = mass of IS added to the test portion, in ng (see Table 7 below)

Table 7. Mass of IS added to the test portion

IS	m_{is} (ng)
^{13}C-AFLAs	5
^{13}C-AFLA M1	5
^{13}C-DON	250
^{13}C-T-2, ^{13}C-HT-2	125
^{13}C-ZEN	100
^{13}C-FB1, ^{13}C-FB2	500
^{13}C-OTA	5

Annex A: Overview of the sample preparation

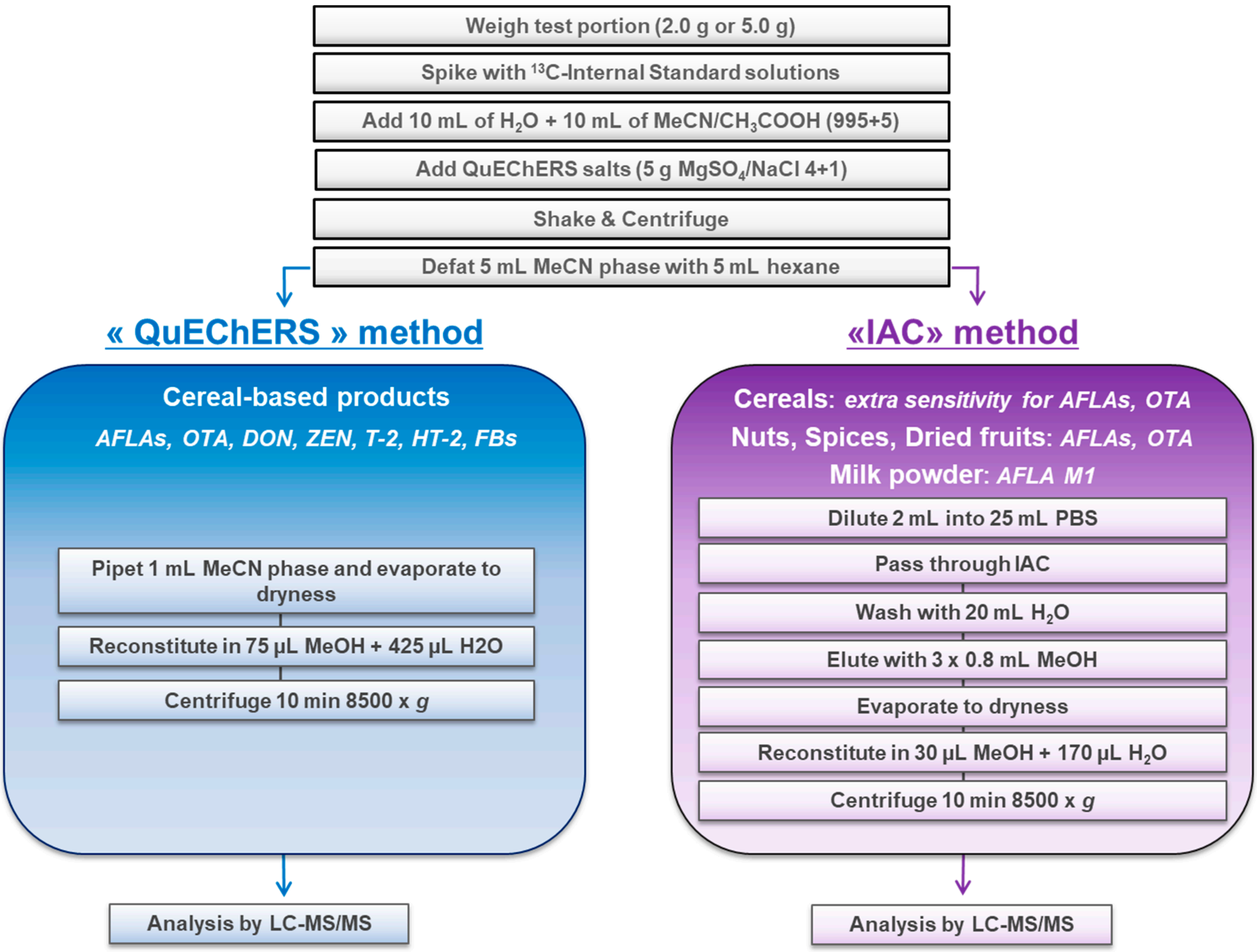

Annex B:
(informative)

Example: Instrumental LC-MS/MS conditions

B.1.1 LC conditions

U(H)PLC system: Agilent 1290
Column: Waters BEH C18 (2.1 x 100 mm, 1.7 µm)
Guard column: Waters BEH C18 Vanguard Pre-column (2.1 mm x 5 mm, 1.8 µm)
Mobile phase A: Formic acid (1.5 mL/L) in water containing 10 mM of ammonium formate
Mobile phase B : Formic acid (0.5 mL/L) in methanol
Injection volume: 10 µL
Temperature: 50 °C

Table A. LC gradient for multi mycotoxin analysis

Time [min]	A [%]	B [%]
0	85	15
0.3	85	15
4	0	100
7	0	100
7.1	85	15
10	85	15

B.1.2 LC conditions 2

HPLC system: Agilent 1260
Column: ZORBAX Bonus-RP (2.1 mm x 150 mm, 3.5 µm)
Guard column: ZORBAX SB-C8 (2.1 mm x 12.5 mm, 5 µm)
Mobile phase A: Formic acid (1.5 mL/L) in water containing 10 mM of ammonium formate
Mobile phase B : Formic acid (0.5 mL/L) in methanol
Injection volume: 10 µL
Temperature: 50 °C

Table B. LC gradient for multi mycotoxin analysis

Time [min]	A [%]	B [%]
0	85	15
0.5	85	15
6.5	0	100
11	0	100
11.5	85	15
19	85	15

B.2 MS conditions

MS system: 6500+ Qtrap (Sciex)
Ionisation: ESI Positive / Negative switching
Ion spray voltage: +5 kV / -4 kV
Source temperature: 550 °C
Gas: CUR: 40 psi; GS1: 40 psi; GS2: 40 psi
CID gas pressure: 10 mTorr (high)

Analyte	Precursor ion	Ionisation state	Product ions	DP*	CE*	EP*	CXP*
**DON**	297.2	$[M+H]^+$	249.2	40	20	10	15
			203.2	40	16	10	15
**($^{13}C_{15}$)-DON**	312.4	$[M+H]^+$	263.2	40	16	10	15
			216.1	40	21	10	15
**AFLAB1**	313.1	$[M+H]^+$	285.1	65	32	10	15
			241.2	65	50	10	15
**($^{13}C_{17}$)-AFLAB1**	330.3	$[M+H]^+$	301.0	50	31	10	15
			255.2	50	50	10	15
**AFLAB2**	315.1	$[M+H]^+$	287.1	65	32	10	15
			259.2	65	40	10	15
**($^{13}C_{17}$)-AFLAB2**	332.0	$[M+H]^+$	303.1	60	35	10	15
			273.1	60	40	10	15
**AFLAG1**	329.1	$[M+H]^+$	243.1	40	37	10	15
			200.0	40	54	10	15
**($^{13}C_{17}$)-AFLAG1**	346.1	$[M+H]^+$	257.2	50	37	10	15
			212.1	50	55	10	15
**AFLAG2**	331.0	$[M+H]^+$	313.1	40	34	10	15
			245.2	40	40	10	15
**($^{13}C_{17}$)-AFLAG2**	348.1	$[M+H]^+$	330.2	50	35	10	15
			259.1	50	42	10	15
**AFLAM1**	329.1	$[M+H]^+$	273.1	30	35	10	15
			229.1	30	57	10	15
**($^{13}C_{17}$)-AFLAM1**	346.2	$[M+H]^+$	288.2	30	35	10	15
			242.3	30	55	10	15
**FB1**	722.4	$[M+H]^+$	334.4	80	55	10	15
			352.4	80	50	10	15
**($^{13}C_{34}$)-FB1**	756.4	$[M+H]^+$	356.4	60	55	10	15
			374.5	60	50	10	15
**FB2**	706.5	$[M+H]^+$	336.3	60	50	10	15
			318.4	60	52	10	15
**($^{13}C_{34}$)-FB2**	740.6	$[M+H]^+$	358.4	60	50	10	15
			340.4	60	53	10	15
**T2**	484.4	$[M+NH_4]^+$	305.0	35	18	10	15
			215.3	35	25	10	15
**($^{13}C_{24}$)-T2**	508.4	$[M+NH_4]^+$	322.2	30	20	10	15
			229.3	30	25	10	15
**HT2**	442.4	$[M+NH_4]^+$	263.3	30	17	10	15
			215.1	30	18	10	15

Analyte	Precursor ion	Ionisation state	Product ions	DP*	CE*	EP*	CXP*
**($^{13}C_{22}$)-HT2**	464.5	$[M+NH_4]^+$	278.3	30	17	10	15
			229.3	30	20	10	15
**ZEN**	317.2	$[M-H]^-$	175.1	-80	-30	10	15
			131.2	-80	-38	10	15
**($^{13}C_{18}$)-ZEN**	335.1	$[M-H]^-$	185.1	-80	-33	10	15
			140.1	-80	-40	10	15
**OTA**	404.2	$[M+H]^+$	239.2	35	30	10	15
			221.1	35	47	10	15
**($^{13}C_{20}$)-OTA**	424.3	$[M+H]^+$	250.2	40	32	10	15
			232.2	40	50	10	15

* DP: declustering potential (V) / CE: collision energy (eV) / EP: entrance potential (V) / CXP: collision exit potential (V).

Bibliography

[1] Commission regulation (EC) No 1881/2006 of 19 December 2006 setting maximum levels for certain contaminants in foodstuffs (Text with EEA relevance).

[2] Commission regulation (EU) No 519/2014 of 16 May 2014 amending Regulation (EC) No 401/2006 as regards methods of sampling of large lots, spices and food supplements, performance criteria for T-2, HT-2 toxin and citrinin and screening methods of analysis (Text with EEA relevance).

[3] Regulation EC No 1907/2006 of the European Parliament and of the Council, *Official Journal of the European Union* Corrigendum in L 136/3 of 29 May 2007, in its current (consolidated) version.

[4] EN 15662:2008 Foods of plant origin - Determination of pesticide residues using GC-MS and/or LC-MS/MS following acetonitrile extraction/partitioning and clean-up by dispersive SPE - QuEChERS-method.

[5] SANTE/12089/2016 Guidance document on identification of mycotoxins in food and feed.

[6] Commission regulation (EC) No 401/2006, of 23 February 2006 *laying down the methods of sampling and analysis for the official control of the levels of mycotoxins in foodstuffs* (Text with EEA relevance).
